# Overexpression of Rice Auxilin-Like Protein, XB21, Induces Necrotic Lesions, up-Regulates Endocytosis-Related Genes, and Confers Enhanced Resistance to *Xanthomonas oryzae* pv. *oryzae*

**DOI:** 10.1186/s12284-017-0166-1

**Published:** 2017-06-02

**Authors:** Chang-Jin Park, Tong Wei, Rita Sharma, Pamela C. Ronald

**Affiliations:** 1 Department of Plant Pathology and the Genome Center, University of California Davis, Davis, CA 95616 USA; 2 Department of Bioresources Engineering and the Plant Engineering Research Institute, Sejong University, Seoul, 05006 Republic of Korea; 3 School of Computational & Integrative Sciences, Jawaharlal Nehru University, New Delhi, 110067 India; 4 Feedstocks Division, Joint BioEnergy Institute, Lawrence Berkeley National Laboratory, Berkeley, CA 94720 USA; 5 Environmental Genomics and Systems Biology, Lawrence Berkeley National Laboratory, Berkeley, CA 94720 USA

**Keywords:** Auxilin, Auxilin-Like Protein, Disease Resistance, Heat Shock Cognate Protein, Heat Shock Protein, XA21 Immune Receptor, XA21 Binding Protein 21, *Xanthomonas oryzae* pv. *oryzae*

## Abstract

**Background:**

The rice immune receptor XA21 confers resistance to the bacterial pathogen, *Xanthomonas oryzae* pv. *oryzae* (*Xoo*). To elucidate the mechanism of XA21-mediated immunity, we previously performed a yeast two-hybrid screening for XA21 interactors and identified XA21 binding protein 21 (XB21).

**Results:**

Here, we report that XB21 is an auxilin-like protein predicted to function in clathrin-mediated endocytosis. We demonstrate an XA21/XB21 in vivo interaction using co-immunoprecipitation in rice. Overexpression of *XB21* in rice variety Kitaake and a Kitaake transgenic line expressing XA21 confers a necrotic lesion phenotype and enhances resistance to *Xoo*. RNA sequencing reveals that *XB21* overexpression results in the differential expression of 8735 genes (4939 genes up- and 3846 genes down-regulated) (≥2-folds, FDR ≤0.01). The up-regulated genes include those predicted to be involved in ‘cell death’ and ‘vesicle-mediated transport’.

**Conclusion:**

These results indicate that XB21 plays a role in the plant immune response and in regulation of cell death. The up-regulation of genes controlling ‘vesicle-mediated transport’ in XB21 overexpression lines is consistent with a functional role for XB21 as an auxilin.

**Electronic supplementary material:**

The online version of this article (doi:10.1186/s12284-017-0166-1) contains supplementary material, which is available to authorized users.

## Background

Protein folding, unfolding, and turnover is central to cell function and is regulated by molecular chaperones, such as heat shock proteins (Hsps) (Saibil 2013). Dysregulation of protein folding can cause cell death and other protein misfolding diseases. Among the major families of HSPs, heat shock cognate protein 70s (Hsc70s) play important roles in protein folding, disaggregation, and transportation. Hsp40, which acts as a co-chaperone of these processes, binds to Hsc70 and stimulates the Hsc70 ATPase activity to stabilize the interaction with substrates (Kampinga and Craig 2010). Hsp40 carries a 70 amino acid signature region, called the J domain, thus Hsp40s are also called J proteins.

J-proteins are classified into three types based on the domain arrangement (Cheetham and Caplan 1998). Type I J-proteins contain an N-terminal J-domain, a glycine-rich region, a zinc-finger domain, and a C-terminal domain. Type II J-proteins are like type I J-proteins except that the zinc-finger domain is missing. Type III J-proteins contain the characteristic J-domain but are structurally divergent outside the J-domain (Koutras and Braun 2014). Type I and II J-proteins are functionally similar in substrates binding, whereas type III J-proteins have distinct roles in stimulating Hsc70 activity due to the flexibility in the structures. One class of type III J-proteins, auxilin and auxilin-like protein (ALP), plays an important role in clathrin-mediated endocytosis. During this process, vesicles are coated by clathrin, a critical component for endocytosis. The clathrin-coated vesicles then transport receptor proteins and their ligands from the cell surface and the trans-Golgi network to the endosomal system (Ungewickell et al. 1995). The J-domain of auxilin recruits Hsc70 to newly budded clathrin-coated vesicles and the Hsc70 and auxilin cooperatively remove the clathrin coat uncoating the vesicles (Kampinga and Craig 2010; Lemmon 2001).

Plant genomes contain more J-proteins than animals. For example, the *Arabidopsis* and rice genomes contains 120 and 125 potential J-proteins respectively, whereas the human genome contains only 41 J-proteins (Sarkar et al. 2013; Rajan and D'Silva 2009). *Arabidopsis*, rice and human encode 92, 83 and 23, type III J-proteins, respectively (Sarkar et al. 2013; Rajan and D'Silva 2009; Qiu et al. 2006), which are the most abundant type of J-proteins. Recent studies of several plant J-proteins suggest that they function in both plant development and the immune response (examples in Sarkar et al. 2013). However, most of the J-proteins have not been characterized and the mechanism of action is unknown.

The rice XA21 immune receptor is representative of a large class of receptor kinases involved in plant innate immunity (Pruitt et al. 2015; Schwessinger and Ronald 2012; Song et al. 1995; Dardick and Ronald 2006). A tyrosine-sulfated peptide from *Xoo*, called RaxX (required for activation of XA21-mediated immunity X), is recognized by XA21 and triggers XA21-mediated immune responses (Pruitt et al. 2015). To elucidate the mechanism of XA21-mediated resistance, we previously performed a yeast two-hybrid screening for XA21 interaction partners using a rice cDNA library (Park et al. 2008; Seo et al. 2011). We previously reported the characterization of four of these XA21 binding proteins including a WRKY transcription factor (Peng et al. 2008), a ubiquitin ligase (Wang et al. 2006), an ATPase (Chen et al. 2010b) and a protein phosphatase 2C (Park et al. 2008).

Here we report the characterization of XA21 binding protein 21 (XB21), predicted to encode a type III J-protein. To determine the in vivo biological function of XB21, we carried out biochemical and transgenic analysis. Co-immunoprecipitation indicates that XB21 directly interacts with the XA21 immune receptor in vivo. Overexpression of *XB21* in rice confers enhanced resistance to *Xoo,* sometimes accompanied with a cell death phenotype. RNA sequencing (RNAseq) analysis of XA21 transgenic plants overexpressing *XB21* indicates that genes related to ‘vesicle-mediated transport’ (which includes genes controlling clathrin-mediated endocytosis) and ‘cell death’ are significantly up-regulated. Taken together, these results indicate that XB21 functions in plant immunity and cell death regulation and suggest that XB21 functions as an auxilin.

## Results

### *XB21* Encodes a Type III J-Protein

**Fig. 1:**
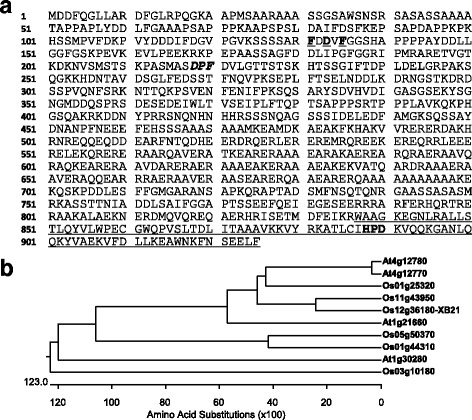
Deduced amino acid sequence of XB21 and phylogenetic analysis of XB21 and type III J-proteins from rice and *Arabidopsis*. **a** The XB21 protein has a C-terminal J-domain similar to auxilin. The auxilin-like-C-terminal domain including a J-domain is underlined. Underlined bolds and bold italic indicate an FxD/NxF motif and a DPF AP-2 binding motif, respectively. **b** Phylogenetic relationships among XB21 and type III J-proteins from rice and *Arabidopsis*. For determining phylogenetic relationships, protein sequences of putative plant proteins related to type III J-proteins were aligned and then used in ClustalW. Ten thousand bootstrap replicates were performed. At4g12780, At4g12770, At1g21660, and At1g30280 from *Arabidopsis*; and Os01g25320, Os11g43950, Os05g50370, Os01g44310, Os03g10180, and Os12g36180 (XB21) from rice

*XB21* contains a predicted 2778 bp open reading frame that encodes a 926 amino acid protein with a molecular weight of 102.0 kDa (Fig. 1a). Phylogenetic analysis with closely related rice and *Arabidopsis* type III J-proteins demonstrates that At4g12770, At4g12780, Os11g43950, Os12g36180-XB21, and Os01g25320 cluster in one group (Fig. 1b). XB21 shows relatively high identity to one rice type III J-proteins (Os11g43950, 63.9% identity) and two *Arabidopsis* type III J-proteins (At4g12770, 50.7% identity and At4g12780, 50.4% identity) (Fig. 1a; Additional file 1: Figure S1).

XB21 contains a conserved J-domain at the C-terminus (837–925 amino acids, underlined in Fig. 1a), and possesses a conserved histidine-proline-aspartic acid (HPD) motif known to interact with Hsc70 (Fig. 1a; Additional file 1: Figure S1). The N-terminal region of XB21 does not contain other recognizable domains. XB21 carries an Asp-Pro-Phe/Trp (DPF/W) adaptor protein-binding motif (bold and italic) (Brett et al. 2002). This motif is predicted to bind to the appendage domain of α- or β-subunits of an adaptor protein complex AP-2, a key component in clathrin-mediated endocytosis. XB21 also contains another Phe-x-Asp/Asn-x-Phe (FxD/NxF; x is any amino acid) motif within the short conserved segment at the N terminus (bold and underlined) (Fig. 1a; Additional file 1: Figure S1). This FxD/NxF motif is predicted to serve as an AP-2 α appendage-binding motif and is found in several accessory proteins indispensable for assembly of clathrin-coated vesicles (Brett et al. 2002). These results indicate that XB21 is a type III J-protein with motifs consistent with a role in clathrin-mediated endocytosis.

### XB21 Interacts With XA21 in Vivo

**Fig. 2:**
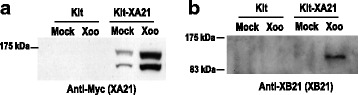
Rice XB21 interacts with XA21 in vivo*.* XB21 co-immunoprecipitated with XA21 protein before (Mock) and after *Xoo* inoculation (*Xoo*) in Kitaake (Kit) and transgenic rice carrying *Myc-XA21* under the control of its native promoter (Myc-XA21, T330-16-1). The precipitates were used for western gel blot analysis using anti-Myc antibody (**a**) or anti-XB21 antibody (**b**). Myc-XA21 and its cleavage product displayed bands at approximately 140 and 100 kDa, respectively, and XB21 was detected as approximately 110 kDa band. Experimental results were repeated three times, with similar results

We previously demonstrated that XB21 and XA21 interact in yeast (Seo et al. 2011). To assess if the proteins interact in vivo, we carried out a co-immunoprecipitation assay. Proteins extracted from Kitaake transgenic rice plants expressing N-terminal Myc-tagged XA21 under the control of its native promoter (Myc-XA21, line T330-16-1) (Park et al. 2008; Park et al. 2010) and the Kitaake control not carrying *XA21* were immunoprecipitated with agarose beads conjugated with anti-Myc antibodies. Western blot analysis of the immunoprecipitated proteins revealed the presence of a 140 kDa polypeptide corresponding to the size of Myc-XA21 in mock treated and *Xoo*-infected Myc-XA21 plants but not from the Kitaake control (Fig. 2a). In the protein sample prepared from the Myc-XA21 infected with *Xoo*, a band of approximately 120 kDa polypeptide was detected with an anti-XB21 antibody (Fig. 2b). The band was not observed in Kitaake and was barely detectable in the mock-treated Myc-XA21 sample. These results demonstrate the *in planta* interaction between XB21 and XA21.

### Ectopic Expression of *XB21* Confers Enhanced Resistance to *Xoo* in Both Kitaake and *XA21* Transgenic Plants

To investigate the biological relevance of *XB21*, we generated transgenic rice lines overexpressing *XB21* (XB21ox/Kit) under the control of the maize ubiquitin promoter in the Kitaake genetic background. Eighteen independent T_0_ transgenic lines overexpressing *XB21* were inoculated with *Xoo*. Six lines (4A, 5A, 7A, 8A, 10A, and 12A) displayed enhanced resistance as compared with the Kitaake control (Additional file 1: Figure S2). We further analyzed XB21ox/Kit T_1_ progenies from 4A, 5A, 7A, 10A, 10B, and 15A, and found that the transgenic plants developed necrotic lesions at the 4 to 5-week stage and became severe at 6-week stage regardless of *Xoo* inoculation (Additional file 1: Figure S3). Six-week-old XB21ox/Kit plants were then inoculated *Xoo*.

**Fig. 3:**
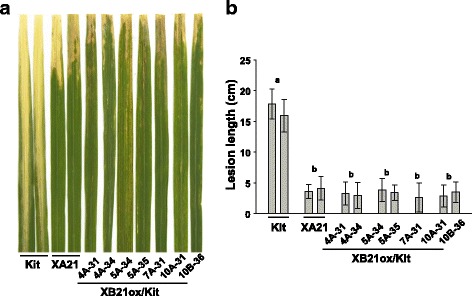
Kitaake wild type rice plants overexpressing *XB21* (XB21ox/Kit) exhibit enhanced resistance to *Xoo*. **a** Rice plants 14 days after inoculation with *Xoo*. From left to right: Kitaake wild type (Kit), transgenic line (XA21, 23A-1-14) carrying *XA21* driven from its native promoter, and transgenic Kitaake lines carrying *XB21ox* construct (XB21ox/Kit). **b** Lesion lengths of *Xoo* inoculated plants of Kitaake wild type (Kit), transgenic line (XA21) carrying *XA21* driven from its native promoter, and transgenic Kitaake lines carrying *XB21ox* construct (XB21ox/Kit)

They exhibited enhanced resistance to *Xoo*, with lesions of approximately 3–5 cm, much shorter than the infected Kitaake plants, which display lesion lengths of approximately 15–20 cm (Fig. 3; Additional file 1: Figure S4). Overexpression of *XB21* was assessed in XB21ox/Kit T_1_ progeny from 4A, 5A and 10B using RT-PCR with *XB21* specific primers (Additional file 1: Figure S5). Transgenic progenies from 4A, 5A, and 10B were displayed high levels of *XB21* transcript compared with Kitaake plants and progenies not carrying *XB21ox* construct. To determine if the enhanced resistance and cell death phenotypes observed in XB21ox/Kit T_1_ are stably transmitted to a third generation, 6-week old XB21ox/Kit plants T_2_ (progeny from 5A-7 and 10A-1) were inoculated with *Xoo*. All T_2_ progenies carrying the XB21ox construct displayed enhanced resistance and necrotic lesions (Additional file 1: Figure S6). These results demonstrate that *XB21* overexpression correlates with the presence of a necrotic lesion phenotype and enhanced resistance to *Xoo*.

**Fig. 4:**
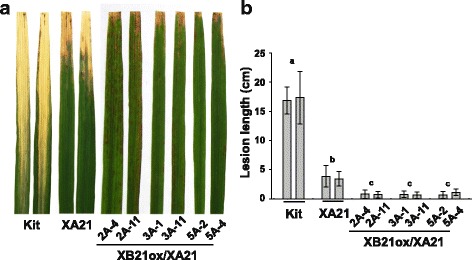
XA21 transgenic rice plants overexpressing *XB21* (XB21ox/XA21) confer enhanced resistance to *Xoo.* **a** Rice plants 12 days after inoculation with *Xoo*. From left to right: Transgenic lines carrying *XB21ox* construct (XB21ox/XA21), transgenic line (XA21, 23A-1-14) carrying *XA21* driven from its native promoter, and Kitaake wild type (Kit). **b** Lesion lengths of *Xoo* inoculated plants of transgenic lines carrying *XB21ox* construct (XB21ox/XA21), transgenic line (XA21) carrying *XA21* driven from its native promoter, and Kitaake wild type (Kit)

To determine if *XB21* overexpression also enhances resistance in the XA21 genetic background, we introduced the XB21ox construct into the transgenic Kitaake carrying *XA21* driven by its native promoter (XA21, line 23A-1-14; described in Chern et al. 2005). We generated 14 independently transformed XB21ox/XA21 T_0_ lines. Of these, six lines (2A, 3A, 5A, 6A, 15A, and 23A) developed necrotic lesions that became more severe as the plants grew older (after 4-weeks) under greenhouse condition. Many T_1_ progenies derived from XB21ox/XA21 transgenic lines 2A, 3A, 5A and 6A displayed necrotic lesions (Additional file 1: Figures S7 and S8) and, exhibited moderate but significant enhanced resistance compared with the XA21 control plants after *Xoo* inoculation, (Fig. 4a and b). The necrotic lesions by XB21 overexpression, however, were not consistently observed in our greenhouse and chamber conditions. The T_1_ progenies from these lines segregated for expression of necrotic lesions and enhanced resistance to *Xoo*. Only the XB21ox/XA21 transgenic plants exhibiting necrotic lesions (black bars in Additional file 1: Figure S8) displayed enhanced resistance to *Xoo.* To investigate whether the necrotic lesions are associated with the expression levels of *XB21*, overexpression of *XB21* was assessed in XB21ox/XA21 T_1_ progeny from 2A, 3A, and 5A using RT-PCR with *XB21* specific primers (Additional file 1: Figure S9). We found no significant difference in *XB21* expression between segregants displaying and not displaying necrotic lesions. Thus, *XB21* overexpression does not correlate with the expression of necrotic lesion in the XA21 genetic background. However, expression of necrotic lesions does correlate with enhanced resistance to *Xoo*.

### Silencing of *XB21* Does not Affect Resistance to *Xoo*

Based on the observation that many of the XB21 overexpressing lines display necrotic lesions and enhanced resistance to *Xoo*, we next investigated whether *XB21* silencing affects resistance in the presence and absence of XA21. For this purpose, a DNA sequence specific to *XB21* that diverges from its closely related Os11g43950 was chosen for *XB21* RNAi construction (Additional file 1: Figure S10). The resulting XB21RNAi construct was introduced into the Kitaake and XA21 genetic backgrounds to generate XB21RNAi/Kit and XB21RNAi/XA21 lines, respectively.

**Fig. 5:**
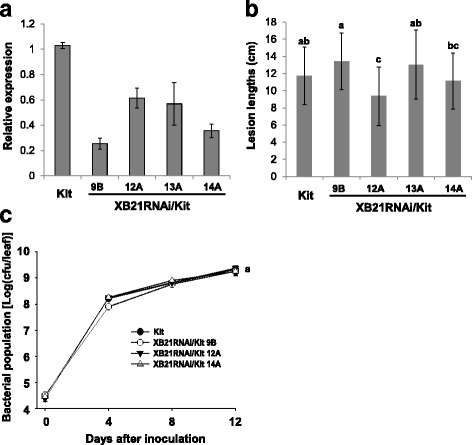
Silencing of XB21 in Kitaake plants does not affect resistance to *Xoo.* **a** Relative expression of *XB21* in Kitaake plants silencing *XB21*. qRT-PCR was performed with specific primers for each genes. Gene expression level was normalized using *ubiquitin* as an internal reference. **b** Lesion lengths measured 14 days after inoculation from Kitaake plants silencing *XB21* (XB21RNAi/Kit 9B, 12A, 13A, and 14A) and Kitaake plants. The error bars represent standard error between three biological replicates. **c** *Xoo* populations were monitored over 12 days in XB21RNAi/Kit (9B, 12A-2, and 14A-2) and Kitaake wild type plants. For each time point, bacterial populations were obtained in three separate leaves for each genotype. The error bars represent standard deviation values obtained from the three samples

Thirteen independently transformed XB21RNAi/Kit T_0_ lines (Additional file 1: Figure S11) were inoculated with *Xoo* and showed no obvious difference in lesion lengths compared with Kitaake control plants inoculated with *Xoo*. Silencing of *XB21* was confirmed in the T_1_ progeny from four T_0_ lines (9B, 12A, 13A, and 14A) by qRT-PCR (Fig. 5a). The progeny from these lines also did not display any significant difference in lesion lengths or bacterial multiplication compared with the Kitaake control (Fig. 5b and c). Additional inoculation experiments on the T_1_ progeny of seven T_0_ lines (3B, 5B, 7A, 8A, 12A, 14A, and 16A) revealed similar results (Additional file 1: Figure S12). These results indicate that silencing of *XB21* expression does not affect resistance to *Xoo* in the Kitaake genetic background.

**Fig. 6:**
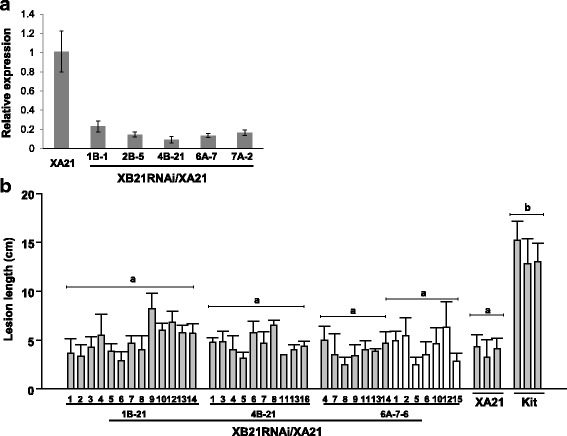
Silencing of XB21 in XA21 transgenic plants does not affect resistance to *Xoo.* **a** Relative expression of *XB21* in XA21 transgenic plants silencing *XB21*. qRT-PCR was performed with specific primers for each genes. Gene expression level was normalized using *ubiquitin* as an internal reference. **b** Lesion lengths measured 12 days after inoculation from XA21 transgenic plants silencing *XB21* (XB21RNAi/XA21 1B-21, 4B-21, and 6A-7-6) and XA21 transgenic plants (23A-1-14). Progeny not carrying *XB21RNAi* construct are labeled with a *white bar*. The error bars represent standard deviation values obtained from the two to seven samples from each plant

We next generated nine independent lines expressing the XB21 RNAi construct in the XA21 genetic background (XB21RNAi/XA21). Silencing of *XB21* was confirmed in the T_1_ progeny from five representative T_0_ lines, 1B, 2B, 4B, 6A and 7A (Fig. 6a). To assess their resistance to *Xoo*, 6-week T_2_ or T_3_ progeny from three XB21RNAi/XA21 lines were inoculated with *Xoo* along with Kitaake and XA21 control plants (Fig. 6b). The progeny do not display significant difference in lesion lengths compared with the XA21 control. In addition, the presence of *XB21ox* construct does not correlated with the lesion lengths in line 6A-7-6. These results suggest that silencing of *XB21* expression does not affect resistance to *Xoo* in the Xa21 genetic background.

### Transcriptomic Analysis Reveals the up-Regulation of Genes Involved in ‘Cell death’ and ‘Vesicle-Mediated transport’ in the XB21ox/XA21 Transgenic Plants

**Fig. 7:**
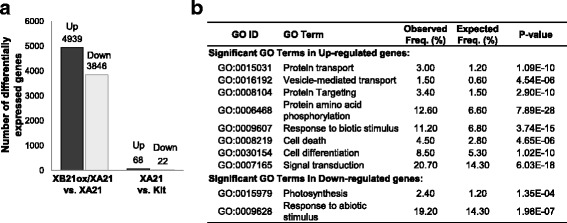
Transcriptional dynamics in response to overexpression of *XB21* in transgenic rice plants carrying *XA21*. **a** Results of differential expression analysis. Numbers of up- and down-regulated genes (≥2-fold change, FDR ≤0.01) in XA21 plants overexpressing *XB21* vs. XA21 plants (XB21ox/XA21 vs. XA21) and XA21 plants vs. Kitaake wild type (XA21 vs. Kit) are presented. **b** Gene Ontology (GO) enrichment analysis for differentially expressed genes (≥2-fold with FDR ≤0.01) performed using Virtual Plant database. Only the top ranked GO terms for “biological process” category are listed. The FDR, calculated using hypergeometric distribution analysis, are provided

To further investigate the mechanism of *XB21* function, we performed a transcriptome analysis of the XB21ox/XA21 line (homozygous progeny derived from the 3A-11 line), the XA21 transgenic line (23A-1-14), and Kitaake controls via RNA-sequencing. Leaf tissue was harvested from five-week-old plants at the point where the XB21ox/XA21 lines began to develop the necrotic lesions. The genes, exhibiting ≥2-fold change in expression with the false discover rate (FDR) ≤0.01, were considered as ‘*differentially expressed*’ and further examined to identify affected pathways. Ninety genes were differentially expressed (≥2-folds, FDR ≤0.01) in the XA21 transgenic plants compared with Kitaake control plants (Fig. 7a; Additional file 2: Table S1A and B). 8735 genes (4939 genes up- and 3846 genes down-regulated) were ‘*differentially expressed*’ in the XB21ox/XA21 plants compared with XA21 plants (Fig. 7a; Additional file 2: Table S1C and D). None of these genes were up-regulated in XA21 plants compared with Kitaake plants indicating that the differential regulation observed in XB21ox/XA21 is dues to XB21 overexpression.

The ‘*differentially expressed*’ genes were used for the Gene Ontology (GO) enrichment analysis (Fig. 7b). The P-value of this analysis revealed that GO terms associated with ‘protein transport’, ‘vesicle-mediated transport’ and ‘protein targeting’ are significantly enriched among the up-regulated genes. Those related with ‘response to biotic stimulus’ and ‘cell death’ are also significantly enriched. Whereas, those regulating ‘photosynthesis’ and ‘response to abiotic stimulus’ were enriched in the down-regulated genes. Because of the necrotic phenotype observed in the XB21ox/XA21 plants and the predicted function of XB21 as an auxilin involved in clathrin-mediated endocytosis based on sequence analysis, we further analyzed genes associated with the GO terms, ‘cell death’ and ‘vesicle-mediated transport’, (Additional file 3: Table S2A and B).

The GO term ‘cell death’ contains 143 differentially expressed genes (Additional file 3: Table S2A). These include up-regulation of putative immune receptors including RGAs (resistance gene analogs) (Sekhwal et al. 2015), RPSs (resistance to *Pseudomonas syringae*) (Axtell and Staskawicz 2003), RPPs (Recognition of *Peronospora parasitica*) (Rehmany et al. 2005), MLOs (powdery mildew locus O) (Piffanelli et al. 2002), and Yr10s (yellow rust resistance protein 10) (Spielmeyer and Lagudah 2003). The differentially regulated genes in the “cell death” set also include Bcl-2 associated athanogene (BAG) domain-containing proteins, which are associated with stress responses and modulation of cell death in humans and *Arabidopsis* (Kabbage and Dickman 2008), chitinases (Jwa et al. 2006), defender against death (DAD family) protein (Danon et al. 2004), BAX inhibitor motif-containing protein, anti-apoptotic protein (Lisak et al. 2015). The up-regulation of the ‘cell death’-related genes in the XB21 overexpression lines correlates with the presence of the necrotic lesions and enhanced resistance observed in the XB21ox/XA21 lines.

We also identified 48 genes with the GO term ‘vesicle-mediated transport’ that are differentially expressed in XB21ox/XA21 (Additional file 3: Table S2B). This set of up-regulated genes include those annotated as exo70 exocyst complex subunits (Wu and Guo 2015), vesicle-associated membrane proteins (Yun et al. 2013), coatomer alpha subunit (Kaur and Subramanian 2015), Sec1 family transport proteins (Karnik et al. 2013), vesicle transport v-soluble N-ethylmaleimide-sensitive factor attachment protein receptor (SNARE) proteins (Bao et al. 2008), adaptins (Park and Jurgens 2011), and component proteins of the clathrin adaptor complex (Qiao et al. 2010). We also identified in this gene set, many clathrin-related protein genes including many clathrin adaptor complex proteins, clathrin assembly proteins, and dynamins (Additional file 2: Table S1 and Additional file 3: Table S2). The up-regulation of genes involved in ‘vesicle-mediated transport’ suggests that XB21 may exert its influence in clathrin-mediated endocytosis.

## Discussion

Here, we characterize a novel XA21-binding protein that carries motifs typical of type III J-protein and is involved in cell death and resistance to *Xoo*.

### XB21 is a Putative Auxilin Protein

XB21 is predicted to encode an auxilin, based on the presence of the C-terminal J domain and the FxD/NxF and DPF/W motifs, which have been found in accessory proteins indispensable for assembly of clathrin-coated vesicles (Lemmon 2001; Brett et al. 2002).

In mammals, there are two highly homologous auxilin-related proteins, auxilin 1 and cyclin-G-dependent kinase/auxilin2 (GAK/auxilin 2). The main structural difference is that GAK/auxilin 2 has an additional N-terminal kinase domain. Despite this structural difference, both auxilin 1 and GAK/auxilin 2 are required for uncoating of clathrin-coated vesicles and for endocytic membrane trafficking in HeLa cells, zebrafish, and *Drosophila* (Hirst et al. 2008; Bai et al. 2010; Kandachar et al. 2008). In *Arabidopsis*, an auxilin-like protein (At4g12780, orthologous gene of rice *XB21*) interacts with plant clathrin and increases clathrin uncoating from microsomal membranes in presence of animal Hsc70 (Lam et al. 2001). These similarities suggest that XB21 also interacts with plant clathrin. In support of this hypothesis, we found that overexpression of XB21 in XA21 rice plants, led to significant induction of genes involved in ‘vesicle-mediated transport’. One of the up-regulated genes, *OsSYP71,* has previously been demonstrated to confer resistance to *Magnaporthe oryzae*, the fungus causing rice blast disease (Bao et al. 2012). *OsSYP71* belongs to the SNARE protein family that functions in vesicle trafficking in eukaryotic cells (Heese et al. 2001; Wick et al. 2003). Taken together, these results suggest that XB21, like mammalian auxilins and *Arabidopsis* auxilin-like proteins, plays a role in uncoating clathrin-coated vesicles.

In animals, a physical interaction between auxilin and cell-surface receptors has not been reported. There are, however, an increasing number of reports showing that auxilin regulates receptor-mediated signaling pathways. For example, *Drosophila* auxilin exhibits specific genetic interactions with Notch receptors, mediating cell-cell interactions during development (Hagedorn et al. 2006). Down-regulation of GAK/auxilin 2 in HeLa cells showed 50-folds *increase* in expression of epidermal growth factor receptor (EGFR), as well as significant changes in downstream EGFR trafficking (Zhang et al. 2004). Depletion of GAK/auxilin 2 in in HeLa cells inhibited receptor-mediated endocytosis and recruitment of clathrin (Lee et al. 2005).

In plants, several reports describing cell-surface receptor endocytosis have been recently published. For example, it has been reported that resistance mediated by *Arabidopsis* Flagellin-sensing 2 (FLS2) immune receptor depends on clathrin-mediated endocytosis (Mbengue et al. 2016). In *Nicotiana benthamiana* silencing *clathrin heavy chain* (*NbCHC*), the endosomal location of heterologously expressed FLS2 was decreased by approximately 75%, indicating defects in the normal endosomal trafficking of FLS2-GFP. XA21 is also reported to be endocytosed and probably transported via trans-Goli network and early endosome compartment (Chen et al. 2010a). Together with these reports, our results suggest that the auxilin-like protein XB21 may function as clathrin uncoating factor to mediate XA21 endocytosis. It is also possible that, considering the general mechanism of clathrin uncoating in the endocytosis process, XB21 could be involved in the endocytosis of other unknown receptors.

### Overexpression of XB21 Causes a Cell Death Phenotype

In animals and plants, cell death occurs during growth and development to remove unnecessary or unwanted cells. In a few cases, mouse and zebrafish, this cell death is mediated by an auxilin (Lee et al. 2008; Bai et al. 2010). In soybean, overexpression of a type III J-protein (GmHsp40.1) caused necrotic cell death (Liu and Whitham 2013). Although, a relationship between auxilin-like proteins and cell death has not been previously observed in plants, the development of cell death lesions in many of the *XB21*-overexpressing plants suggests that XB21 is at least partially involved in this cell death phenotype. We have noted, however, that the XB21-induced cell death is not consistently observed. For example during winter greenhouse trials of the T_1_ progeny of XB21ox/XA21 plants, more than half of the segregating progeny carrying *XB21ox* construct did not display a necrotic lesion phenotype. These results suggest that the developmentally regulated cell death observed in some *XB21* overexpressing plants is also regulated by additional, unidentified abiotic factors.

Several studies have reported a role of vesicular trafficking in plant cell death. For example, rice spotted leaf 28 (*SPL28*) encoding a clathrin-associated adaptor protein complex 1, medium subunit μ1 (AP1M1), which is involved in the post-Golgi trafficking pathway (Qiao et al. 2010). The *spl28* mutants displayed a necrotic lesion phenotype spontaneously in the absence of pathogen attack. Another example is the discovery of the μ subunit of *Arabidopsis* adaptor protein-2 (AP2M), mediating clathrin-coated vesicle formation, is also involved in the necrotic lesion formation. An *ap2m* mutant displayed reduced amount of cell death after *Pseudomonas syringae* pv. *tomato* DC3000 (Hatsugai et al. 2016). A third example is *Arabidopsis* enhanced disease resistance 4 (EDR4) negatively regulating resistance to powdery mildew. The EDR4 interacts with clathrin heavy chain 2 (CHC2) (Wu et al. 2015). The *edr4* mutants displayed cell death and reduced endocytosis rates, indicating that clathrin-mediated membrane trafficking is involved in cell deaths. The molecular mechanisms of how these proteins regulate cell death have not yet been elucidated.

Considering that XB21 possesses a DPF/W motif and a FxD/NxF motif predicted to interact with adaptor protein complex indispensable for clathrin-coated vesicle formation, we hypothesize that XB21 cooperatively works with adaptor proteins and/or clathrins. Supporting this hypothesis, RNAseq analysis in XB21ox/XA21 transgenic plants displayed up-regulation of many clathrin adaptor complex proteins, clathrin assembly proteins and dynamins as well as other genes involved in ‘vesicle-mediated transport’.

## Conclusions

XB21 was originally isolated as an XA21 binding protein in yeast. Here we demonstrate an in vivo interaction between XB21 and XA21 using co-immunoprecipitation assays in rice plants. RNAseq analysis of *XB21*-overexpressing rice plants revealed up-regulation of genes related to diverse biological processes including those regulating ‘vesicle-mediated transport’ and ‘cell death’. These results together with the predicted domain structure of XB21 support the hypothesis that XB21 functions as an auxilin.

## Methods

### Plant Materials and Growth Conditions

*Oryza sativa* L. spp. *japonica* rice variety Kitaake plants (lacking *XA21*) and transgenic plants were maintained in the greenhouse facility at University of California, Davis. The growth chamber for inoculation experiments was set on a 16-h light and 8-h dark period, 28/26 °C temperature cycle and 90% humidity. Healthy and well-expanded leaves from six-week-old plants were used for *Xoo* inoculation as well as nucleic acid and protein extractions.

### In Vivo Co-Immunoprecipitation

To co-immunoprecipitate Myc-XA21 and XB21, total proteins were extracted from 5 g of rice leaf tissue in 25 ml of ice-cold Extraction Buffer [0.15 M NaCl, 0.01 M Na-phosphate pH 7.2, 2 mM EDTA, 10 mM β-mercaptoethanol, 1% Triton X-100, 1 mM PMSF, 20 mM NaF, 1% Protease cocktail (Sigma), 2 μg/ml antipain, 2 μg/ml leupeptin, and 2 μg/ml aprotinin] as described earlier (Park et al. 2008). After filtering through Miracloth (Calbiochem) followed by centrifugation at 13,000 g for 20 min at 4 °C, 50 μl of agarose conjugated anti-Myc antibody (Santa Cruz) was added to the supernatant and incubated at 4 °C for 2 h. The agarose beads were then washed three times in 1.5 ml of extraction buffer without proteinase inhibitors. The immunoprecipitated proteins were eluted with 4× Laemmli sample buffer. Western blot analyses were performed as previously (Park et al. 2008). The anti-XB21 antibody was generated by Pacific Immunology based on a synthetic peptide of XB21. Detailed information about their methods can be obtained from their website (http://www.pacificimmunology.com/). In brief, a short peptide (KPEVKEVLPEEKRKPEPA, 156–173 a.a of XB21) was synthesized and conjugated to an immune-stimulating carrier protein, keyhole limpet hemocyanin. Anti-XB21 antibody was affinity-purified using the synthetic peptides bound to C3-SEP-PAK cartridges.

### *Xoo* Inoculations and Determination of Bacterial Populations

*Xanthomonas oryzae* pv. *oryzae* (*Xoo*) Philippine race 6 (PR6) strain PXO99Az (generously provided by Jan Leach; referred to as *Xoo* throughout text) was used in this study. For *Xoo* inoculations, 6-week old rice plants were transferred to the growth chamber. *Xoo* suspensions (OD600 of 0.5) were used to inoculate rice plants by the scissors-dip method (Song et al. 1995; Chern et al. 2005). Only the top two to three fully expanded leaves of each tiller were inoculated. Lesion lengths were measured at the indicated days after inoculation. Statistical analysis was performed using the Tukey’s honest significant difference test (Tukey’s HSD test). For bacterial colony counts, the inoculated leaves, including lesions and tissue showing no lesions, were cut into approximately 1 mm pieces with sterile scissors and incubated in 10 mL water for 1 h to harvest bacteria. The extract was diluted accordingly and plated on peptone sucrose agar (PSA) plates containing 20 mg/L of cephalexin. The plates were scored after incubation at 37 °C for two days.

### Plasmid Construction for *XB21* Overexpression and Silencing

A 2778 nt cDNA fragment encoding full-length XB21 protein was amplified from rice cDNA using primers, 5´-CACCATGGACGACTTCCAGGGCCTCCTGGCC-3´ and 5´-TTAGAAGAGTTCCTCTGAGTTGAATTTG-3´. The PCR product was cloned in pENTR^TM^/D-TOPO® according to the manufacturer’s instruction (Invitrogen) and the insert was confirmed by Sanger sequencing. For overexpression in rice, the *XB21* cDNA in pENTR^TM^/D-TOPO® was recombined into an Ubi-1300 vector using Gateway® LR Clonase (Invitrogen). Ubi-1300 is a pCAMBIA-1300 (AF234296) derivative containing an additional expression cassette with the maize ubiquitin promoter (Ubi) and a nopaline synthase 3´-polyadenylation region (NOS), to which the Gateway® cassette was added (Rohila et al. 2006).

For *XB21* silencing, a 427 nt cDNA fragment from 529 to 955 bp of *XB21* cDNA was amplified from rice cDNA using *XB21* specific primers, 5´-CACCTCGGCTGGGTTTGATGATTT-3´ and 5´-CAGAAGGTTTTTGGGTTGTATTTT-3´. The cDNA fragment was chosen because of its high diversity with other *XB21* paralogs. The PCR product was cloned in pENTR^TM^/D-TOPO® (Invitrogen) and the insert was confirmed by sequencing. For silencing in rice, the partial *XB21* cDNA fragment in pENTR^TM^/D-TOPO® was recombined into the pANDA silencing vector (Miki and Shimamoto 2004) using gateway LR Clonase (Invitrogen).

### Rice Transformations

Rice transformations were carried out as described previously (Chern et al. 2005). *Agrobacterium tumefaciens* strain EHA105 was used to infect rice callus. Transformants of rice cultivar Kitaake and transgenic XA21 plants (23A-1-14) (referred to as XA21 in this paper) (Park et al. 2010; Chern et al. 2005) carrying *XB21ox* or *XB21RNAi* construct were selected using hygromycin and confirmed by PCR using gene-specific primers.

### Quantitative Real-Time PCR Analysis

For quantitative real time PCR (qRT-PCR) analysis, we harvested fully expended leaves from wild type and transgenic plants and extracted RNA using TRIzol reagent (Invitogen) as per manufacturer’s instructions. Total RNA was treated with DNase I and purified using Macherey-Nagel NucleoSpin RNA II kit. We quantified RNA using Nanodrop (ND-1000 spectrophotomter from Thermo Scientific) and performed cDNA synthesis using superscript VILO cDNA synthesis kit (Invitrogen). About 100 ng of cDNA was used as template for each qRT-PCR reaction using SsoFast EvaGreen Supermix (Bios-Rad). All PCR reactions were performed in 96-well plates on a Bio-Rad CFX96 Real-Time System coupled with a C1000 Thermal Cycler. The expression of target genes was normalized to the internal reference *ubiquitin*. The primers used for qRT-PCR were, 5´- AACCAGCTGAGGCCCAAGA-3´/5´-ACGATTGATTTAACCAGTCCATGA-3´ (for *ubiquitin*), 5´- AAATCCCTGCCTCAGTTGCT-3´/5´-AACCCCGAGGTGATAATTCC-3´ (for *XA21*) and 5´- GGCTGCTGTAGACGCTAG-3´/5´-ACCTTCTCCTTGGCTTCAG-3´ (for *XB21*).

### RNA Sequencing and Analysis

Three replicates, with each replicate of three leaf tissues, were collected from five-week-old Kitaake, transgenic plants carrying *XA21* (XA21, 23A-1-14) and transgenic plants overexpressing *XB21* in the *XA21* genetic background (XB21ox/XA21, progeny of homozygous 3A-11 line), with three replicates. RNA was isolated using TRIzol reagent, DNase-treated and purified using Qiagen RNeasy Plant mini kit. A total of nine cDNA libraries were constructed using Illumina reagents according to manufacturer’s instructions. About 20 million reads were sequenced from each library by U.S. Department of Energy (DOE) Joint Genome Institute (JGI) using Illumina paired-end sequencing (2X76 bp). The high quality reads were mapped to rice reference sequence (MSU 6.1) using Tophat 3.1 (Trapnell et al. 2009), with default parameters. The mapped RNA sequencing (RNAseq) reads were assembled into transcripts by cufflinks (Trapnell et al. 2010). The data was analyzed in RobinA (http://mapman.gabipd.org/web/guest/robin) for differential expression (Lohse et al. 2012). The genes exhibiting ≥2-fold change in expression with FDR ≤0.01 in three biological replicates were defined as ‘*differentially expressed*’ and used for the Gene Ontology (GO) enrichment analysis. The ‘*differentially expressed*’ genes were categorized into pathways using Virtual Plant 1.3 (Katari et al. 2010) and mapped onto functional categories in the MapMan (Thimm et al. 2004).

## Additional files

Additional file 1: Figure S1. Amino acids sequence alignment of ALPs from rice (XB21-Os12g36180 and Os11g43950) and *Arabidopsis* (At4g12770 and At4g12780). Dashes indicate gaps. **Figure S2.** Overexpression of *XB21* in Kitaake wild type plants exhibit enhanced resistance to *Xoo*. **Figure S3.** Kitaake plants overexpressing XB21 (XB21ox/Kit) display cell death lesions. **Figure S4.** T_1_ generation of XB21ox/Kit carrying *XB21ox* construct display enhanced resistance to *Xoo*. **Figure S5.** Transgenic Kitaake plants carrying *XB21ox* construct (XB21ox/Kit) overexpress *XB21*. **Figure S6.** Overexpression of *XB21* in Kitaake wild type plants (XB21ox/Kit, T_2_) displays resistant to a virulent strain of *Xoo*. **Figure S7.** Overexpression of *XB21* in XA21 plants (XB21ox/XA21) displays cell death lesions. **Figure S8.** T1 progeny from XB21 overexpressing XA21 transgenic plants (XB21ox/XA21) display cell death and enhanced resistance to *Xoo*. **Figure S9.** XA21 plants carrying *XB21ox* construct (XB21ox/XA21) overexpress *XB21*. **Figure S10.** Comparison of partial nucleotide sequence used for *XB21* RNA silencing and one of its closely related putative ALP Os11g43950. **Figure S11.** Silencing of *XB21* in Kitaake plants does not exhibit significant difference in lesion lengths after *Xoo* inoculation. **Figure S12.** T_1_ generation of XB21RNAi/Kit do not display alteration of resistance to *Xoo*. (PPTX 3450 kb)

Additional file 2: Table S1. RNA sequencing-mediated transcriptome analysis of the Kitaake wild type, transgenic plants carrying *XA21* (XA21) (23A-1-14), and transgenic plants overexpressing *XB21* in the *XA21* genetic background (XB21ox/XA21, progeny of 3A-11). The genes, exhibiting ≥2-fold change in expression with FDR ≤0.01, were considered as ‘*differentially expressed*’ and listed. (A) Up-regulated genes in XA21 plants compared to Kitaake plants. (B) down-regulated genes in XA21 plants compared to Kitaake plants. (C) Up-regulated genes in XB21ox/XA21 plants compared to XA21 plants. (D) Down-regulated genes in XB21ox/XA21 plants compared to XA21 plants. (XLSX 470 kb)

Additional file 3: Table S2. List of up-regulated genes associated with GO terms, 'cell death' (A) and 'vesicle-mediated transport' (B) in XB21ox/XA21 plants compared to XA21 plants. The genes, exhibiting ≥2-fold change in expression with *p* value ≤0.01, were considered as ‘*differentially expressed*’ and listed. (XLSX 19 kb)

## Abbreviations

Hsc: Heat shock cognate protein
Hsp: Heat shock protein
XB21: XA21 Binding Protein 21
*Xoo* PR6: *Xanthomonas oryzae* pv. *oryzae* Philippine Race 6
