# Supplementary material for: Overexpression of Rice Auxilin-Like Protein, XB21, Induces Necrotic Lesions, up-Regulates Endocytosis-Related Genes, and Confers Enhanced Resistance to Xanthomonas oryzae pv. oryzae
Source: Rice (N Y). 2017 Jun 2;10:27. doi: 10.1186/s12284-017-0166-1 (PMC5457384; doi:10.1186/s12284-017-0166-1)
Supplement: Supplementary file 1 — Amino acids sequence alignment of ALPs from rice (XB21-Os12g36180 and Os11g43950) and Arabidopsis (At4g12770 and At4g12780). Dashes indicate gaps. Figure S2. Overexpression of XB21 in Kitaake wild type plants exhibit enhanced resistance to Xoo. Figure S3. Kitaake plants overexpressing XB21 (XB21ox/Kit) display cell death lesions. Figure S4. T1 generation of XB21ox/Kit carrying XB21ox construct display enhanced resistance to Xoo. Figure S5. Transgenic Kitaake plants carrying XB21ox construct (XB21ox/Kit) overexpress XB21. Figure S6. Overexpression of XB21 in Kitaake wild type plants (XB21ox/Kit, T2) displays resistant to a virulent strain of Xoo. Figure S7. Overexpression of XB21 in XA21 plants (XB21ox/XA21) displays cell death lesions. Figure S8. T1 progeny from XB21 overexpressing XA21 transgenic plants (XB21ox/XA21) display cell death and enhanced resistance to Xoo. Figure S9. XA21 plants carrying XB21ox construct (XB21ox/XA21) overexpress XB21. Figure S10. Comparison of partial nucleotide sequence used for XB21 RNA silencing and one of its closely related putative ALP Os11g43950. Figure S11. Silencing of XB21 in Kitaake plants does not exhibit significant difference in lesion lengths after Xoo inoculation. Figure S12. T1 generation of XB21RNAi/Kit do not display alteration of resistance to Xoo. (PPTX 3450 kb) [file 12284_2017_166_MOESM1_ESM.pptx]

## Slide 1
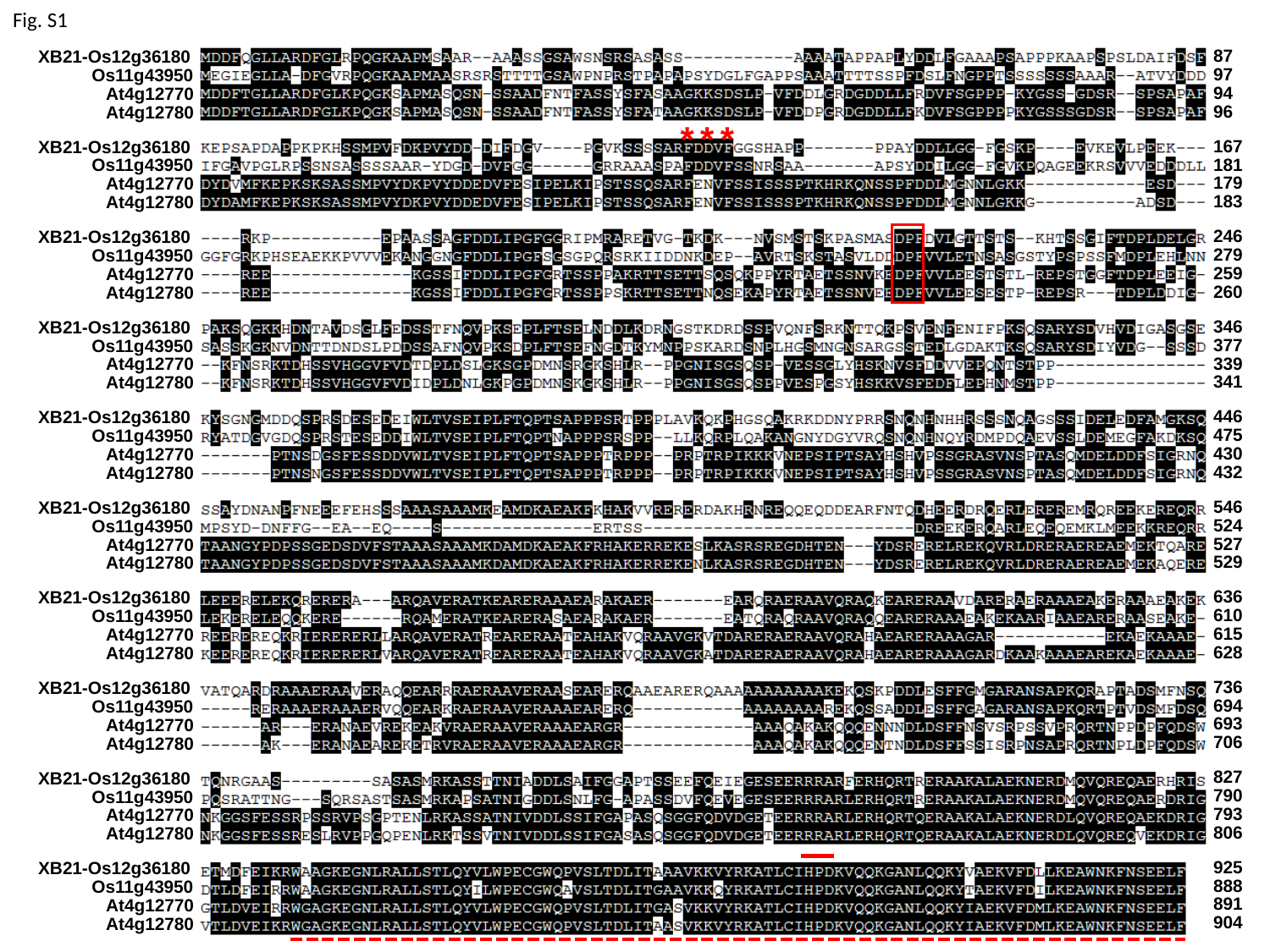

Fig. S1
87
97
94
96
XB21-Os12g36180
Os11g43950
At4g12770
At4g12780
*
*
*
167
181
179
183
XB21-Os12g36180
Os11g43950
At4g12770
At4g12780
246
279
259
260
XB21-Os12g36180
Os11g43950
At4g12770
At4g12780
346
377
339
341
XB21-Os12g36180
Os11g43950
At4g12770
At4g12780
446
475
430
432
XB21-Os12g36180
Os11g43950
At4g12770
At4g12780
546
524
527
529
XB21-Os12g36180
Os11g43950
At4g12770
At4g12780
636
610
615
628
XB21-Os12g36180
Os11g43950
At4g12770
At4g12780
736
694
693
706
XB21-Os12g36180
Os11g43950
At4g12770
At4g12780
827
790
793
806
XB21-Os12g36180
Os11g43950
At4g12770
At4g12780
925
888
891
904
XB21-Os12g36180
Os11g43950
At4g12770
At4g12780

## Slide 2
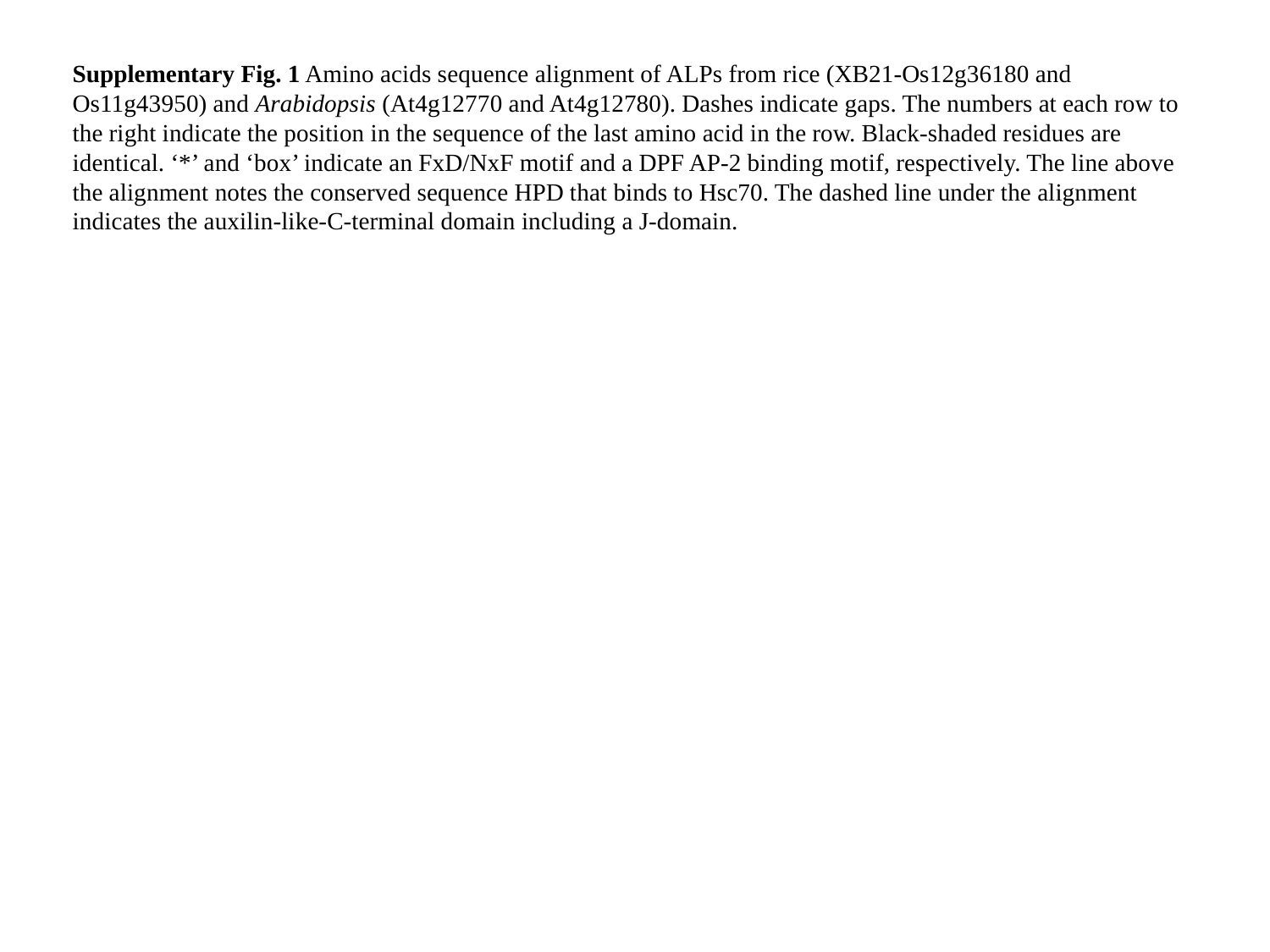

Supplementary Fig. 1 Amino acids sequence alignment of ALPs from rice (XB21-Os12g36180 and Os11g43950) and Arabidopsis (At4g12770 and At4g12780). Dashes indicate gaps. The numbers at each row to the right indicate the position in the sequence of the last amino acid in the row. Black-shaded residues are identical. ‘*’ and ‘box’ indicate an FxD/NxF motif and a DPF AP-2 binding motif, respectively. The line above the alignment notes the conserved sequence HPD that binds to Hsc70. The dashed line under the alignment indicates the auxilin-like-C-terminal domain including a J-domain.

## Slide 3
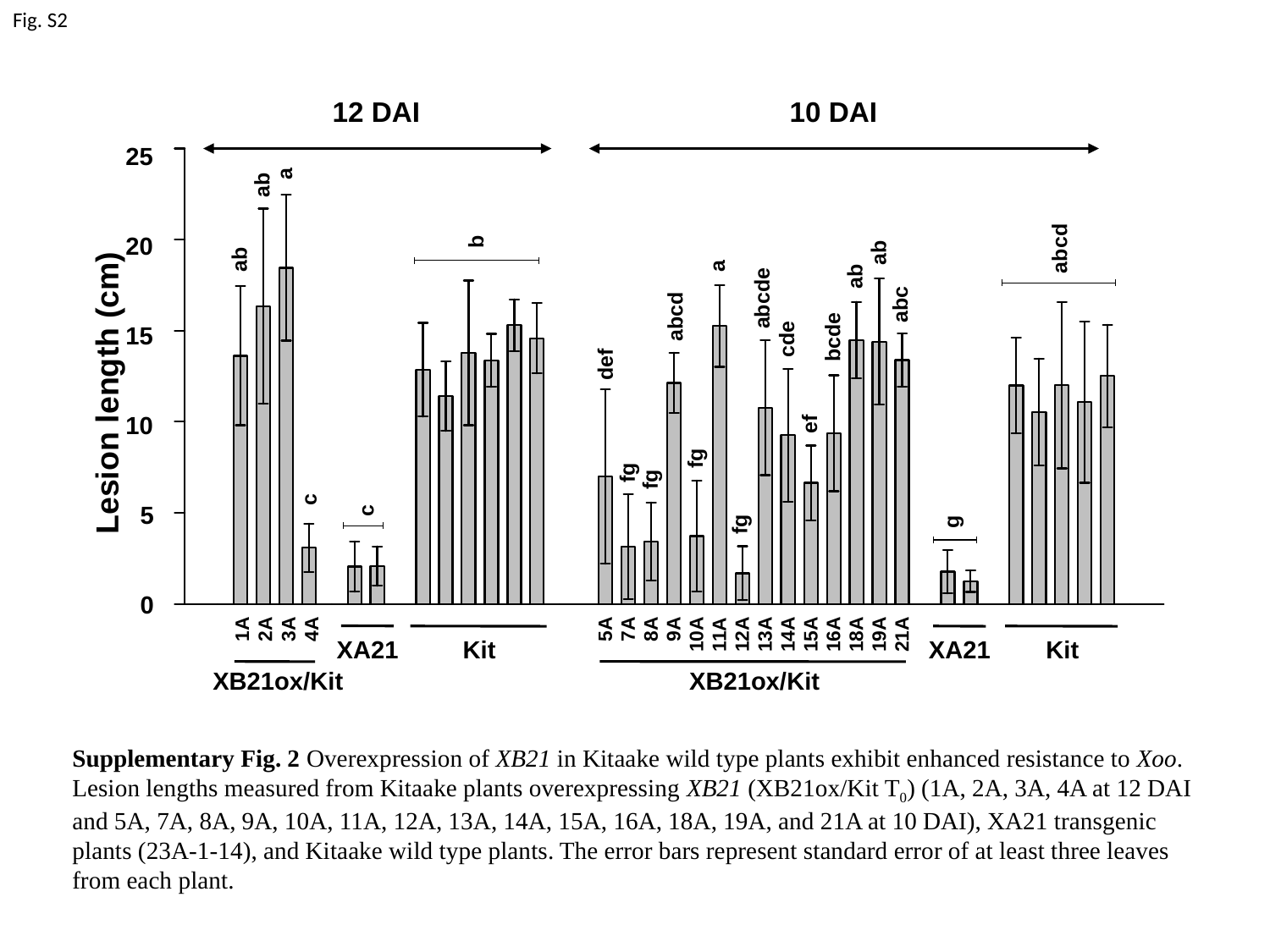

Fig. S2
10 DAI
12 DAI
25
a
ab
b
20
abcd
ab
ab
a
ab
abcde
abc
abcd
15
bcde
cde
def
Lesion length (cm)
10
ef
fg
fg
fg
c
c
5
g
fg
0
1A
2A
3A
4A
5A
7A
8A
9A
10A
11A
12A
13A
14A
15A
16A
18A
19A
21A
XA21
Kit
XA21
Kit
XB21ox/Kit
XB21ox/Kit
Supplementary Fig. 2 Overexpression of XB21 in Kitaake wild type plants exhibit enhanced resistance to Xoo.
Lesion lengths measured from Kitaake plants overexpressing XB21 (XB21ox/Kit T0) (1A, 2A, 3A, 4A at 12 DAI and 5A, 7A, 8A, 9A, 10A, 11A, 12A, 13A, 14A, 15A, 16A, 18A, 19A, and 21A at 10 DAI), XA21 transgenic plants (23A-1-14), and Kitaake wild type plants. The error bars represent standard error of at least three leaves from each plant.

## Slide 4
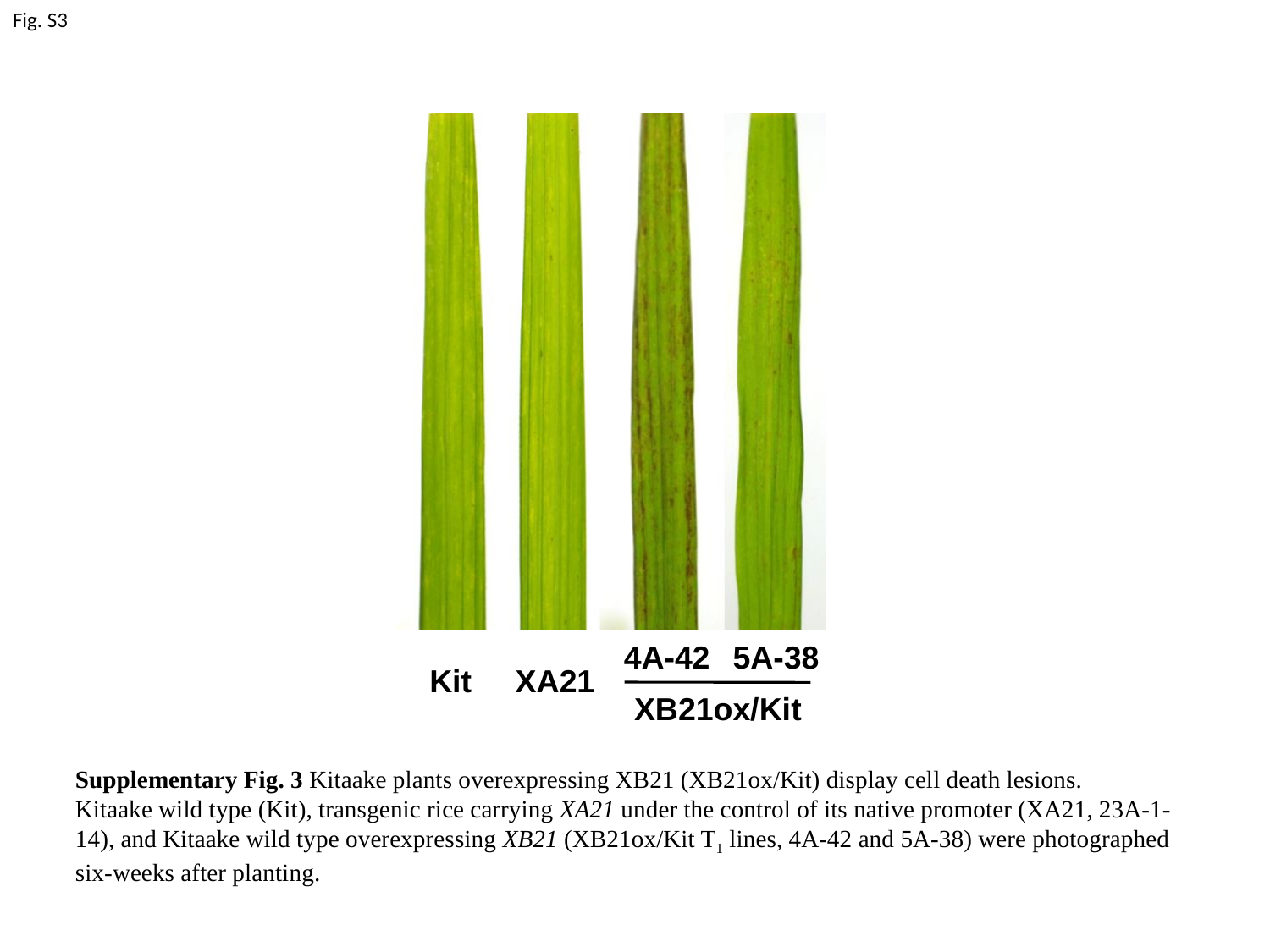

Fig. S3
4A-42
5A-38
Kit
XA21
XB21ox/Kit
Supplementary Fig. 3 Kitaake plants overexpressing XB21 (XB21ox/Kit) display cell death lesions.
Kitaake wild type (Kit), transgenic rice carrying XA21 under the control of its native promoter (XA21, 23A-1-14), and Kitaake wild type overexpressing XB21 (XB21ox/Kit T1 lines, 4A-42 and 5A-38) were photographed six-weeks after planting.

## Slide 5
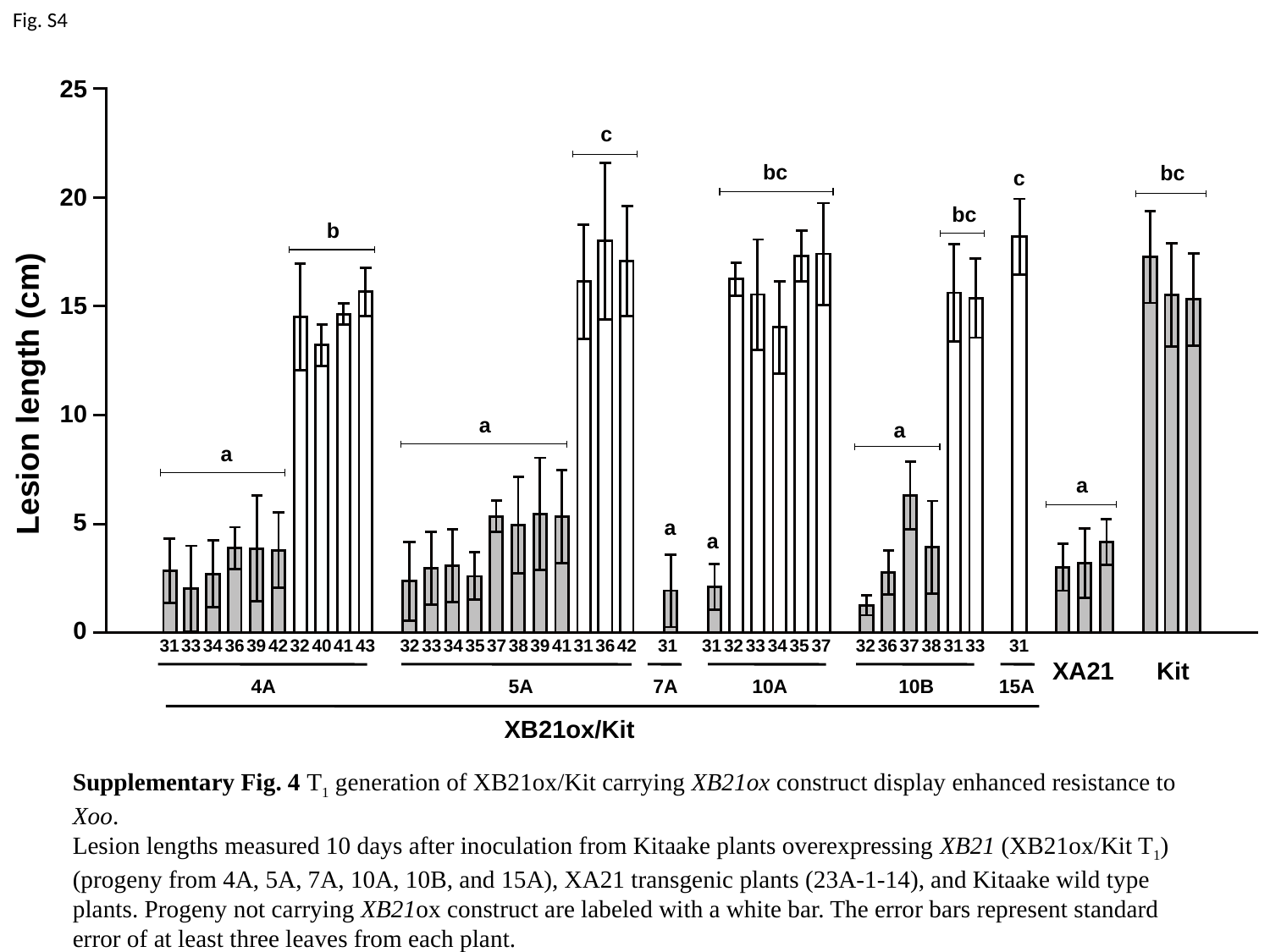

Fig. S4
25
c
bc
bc
c
20
bc
b
15
Lesion length (cm)
10
a
a
a
a
5
a
a
0
31
33
34
36
39
42
32
40
41
43
32
33
34
35
37
38
39
41
31
36
42
31
31
32
33
34
35
37
32
36
37
38
31
33
31
XA21
Kit
4A
5A
7A
10A
10B
15A
XB21ox/Kit
Supplementary Fig. 4 T1 generation of XB21ox/Kit carrying XB21ox construct display enhanced resistance to Xoo.
Lesion lengths measured 10 days after inoculation from Kitaake plants overexpressing XB21 (XB21ox/Kit T1) (progeny from 4A, 5A, 7A, 10A, 10B, and 15A), XA21 transgenic plants (23A-1-14), and Kitaake wild type plants. Progeny not carrying XB21ox construct are labeled with a white bar. The error bars represent standard error of at least three leaves from each plant.

## Slide 6
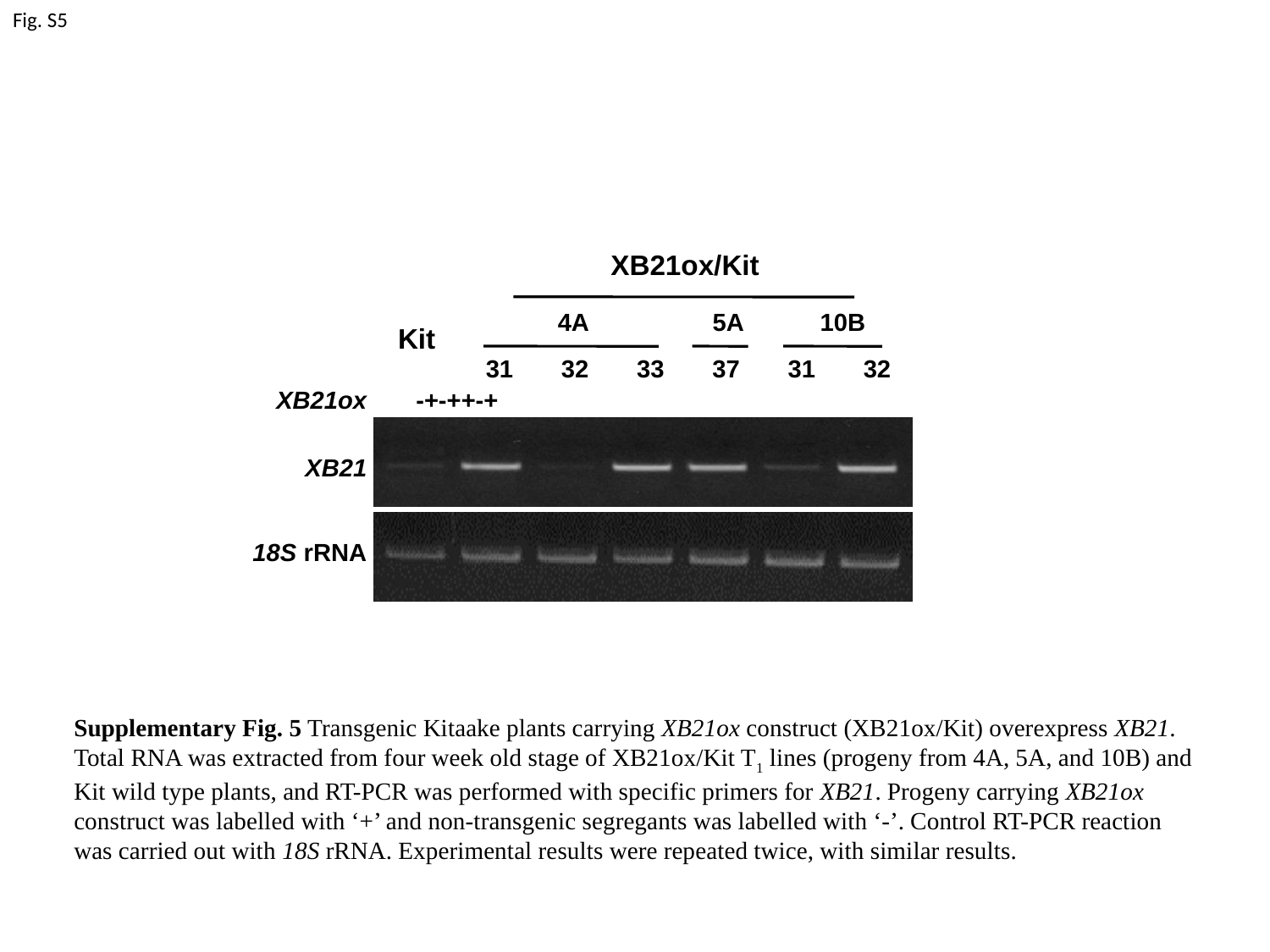

Fig. S5
XB21ox/Kit
4A
5A
10B
Kit
31
32
33
37
31
32
XB21ox
-+-++-+
XB21
18S rRNA
Supplementary Fig. 5 Transgenic Kitaake plants carrying XB21ox construct (XB21ox/Kit) overexpress XB21.
Total RNA was extracted from four week old stage of XB21ox/Kit T1 lines (progeny from 4A, 5A, and 10B) and Kit wild type plants, and RT-PCR was performed with specific primers for XB21. Progeny carrying XB21ox construct was labelled with ‘+’ and non-transgenic segregants was labelled with ‘-’. Control RT-PCR reaction was carried out with 18S rRNA. Experimental results were repeated twice, with similar results.

## Slide 7
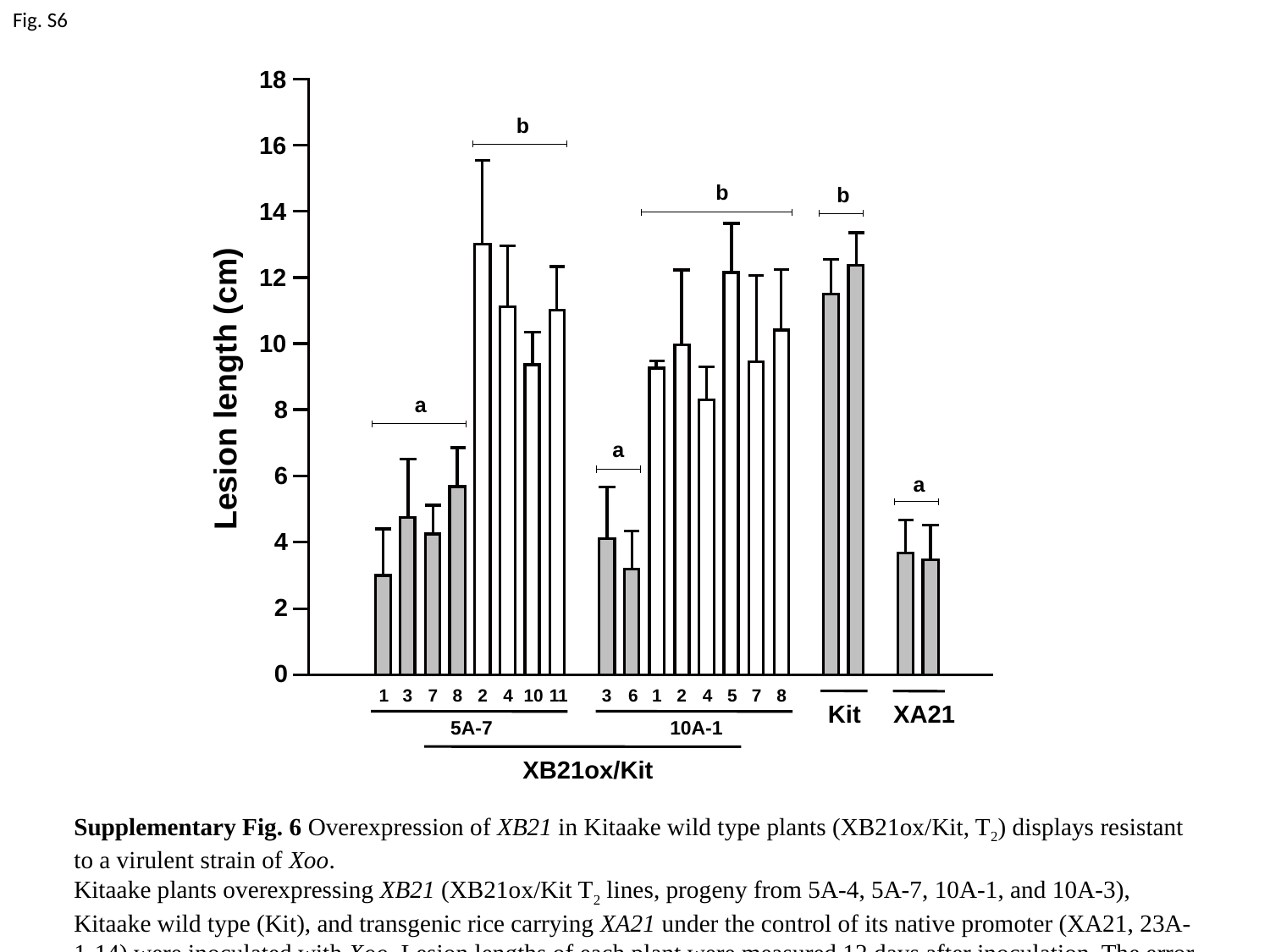

Fig. S6
18
b
16
b
b
14
12
10
Lesion length (cm)
a
8
a
6
a
4
2
0
1
3
7
8
2
4
10
11
3
6
1
2
4
5
7
8
Kit
XA21
5A-7
10A-1
XB21ox/Kit
Supplementary Fig. 6 Overexpression of XB21 in Kitaake wild type plants (XB21ox/Kit, T2) displays resistant to a virulent strain of Xoo.
Kitaake plants overexpressing XB21 (XB21ox/Kit T2 lines, progeny from 5A-4, 5A-7, 10A-1, and 10A-3), Kitaake wild type (Kit), and transgenic rice carrying XA21 under the control of its native promoter (XA21, 23A-1-14) were inoculated with Xoo. Lesion lengths of each plant were measured 12 days after inoculation. The error bars represent standard error of two to five leaves from each plant. Gray bar, progeny carrying XB21ox construct in XB21ox/Kit plants; White bar, progeny not carrying XB21ox construct

## Slide 8
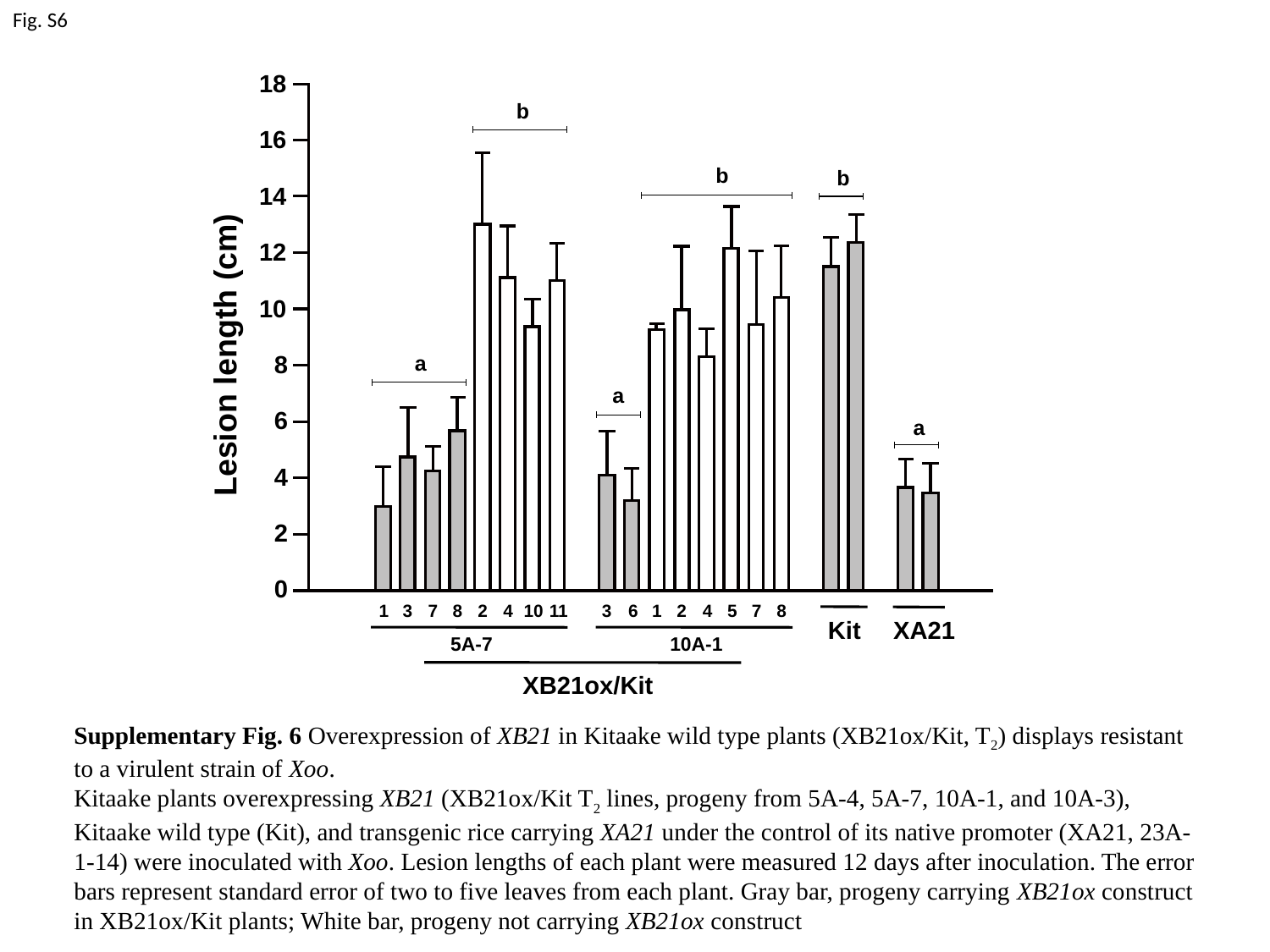

Fig. S6
18
b
16
b
b
14
12
10
Lesion length (cm)
8
a
a
6
a
4
2
0
1
3
7
8
2
4
10
11
3
6
1
2
4
5
7
8
Kit
XA21
5A-7
10A-1
XB21ox/Kit
Supplementary Fig. 6 Overexpression of XB21 in Kitaake wild type plants (XB21ox/Kit, T2) displays resistant to a virulent strain of Xoo.
Kitaake plants overexpressing XB21 (XB21ox/Kit T2 lines, progeny from 5A-4, 5A-7, 10A-1, and 10A-3), Kitaake wild type (Kit), and transgenic rice carrying XA21 under the control of its native promoter (XA21, 23A-1-14) were inoculated with Xoo. Lesion lengths of each plant were measured 12 days after inoculation. The error bars represent standard error of two to five leaves from each plant. Gray bar, progeny carrying XB21ox construct in XB21ox/Kit plants; White bar, progeny not carrying XB21ox construct

## Slide 9
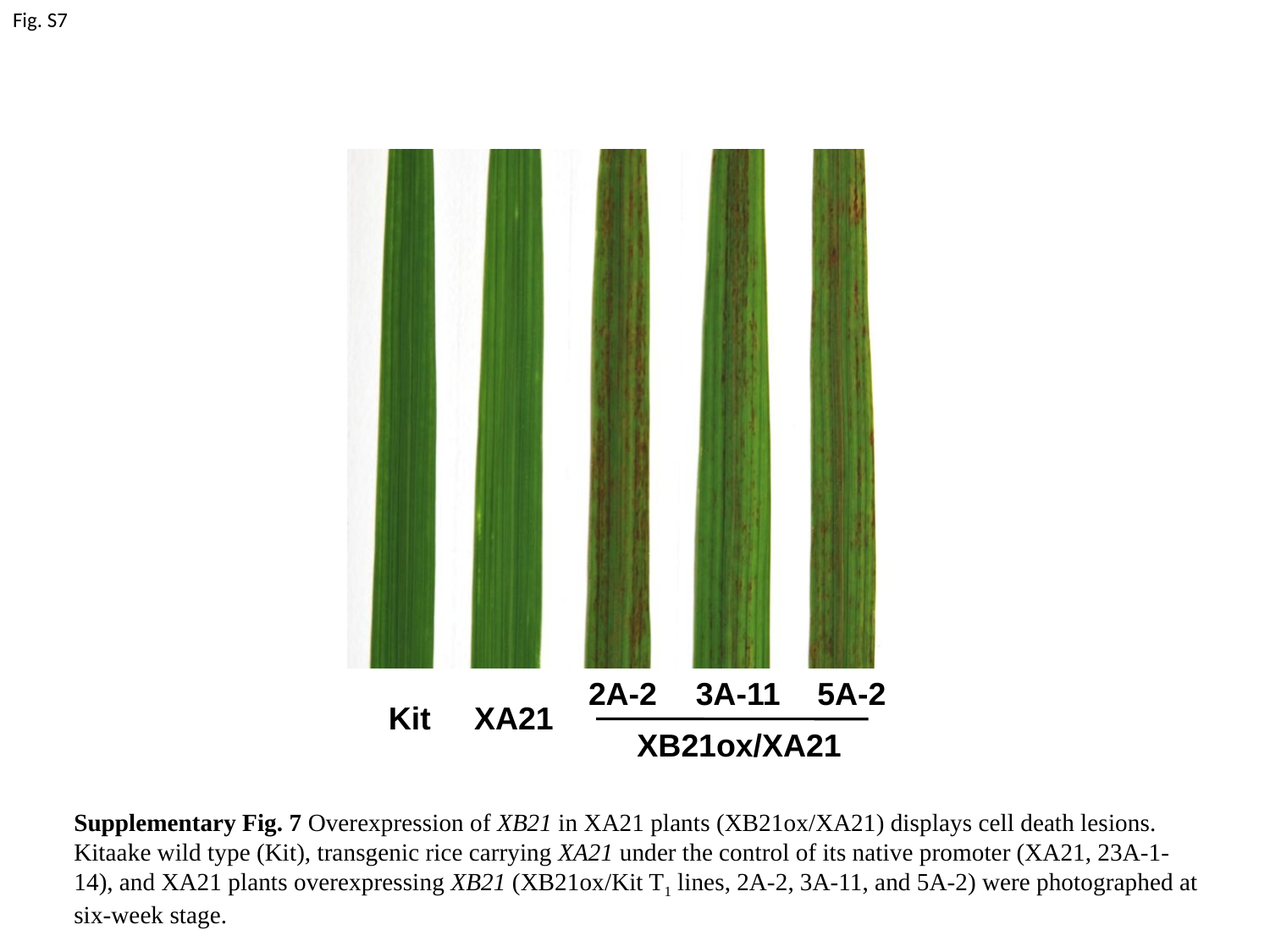

Fig. S7
2A-2
3A-11
5A-2
Kit
XA21
XB21ox/XA21
Supplementary Fig. 7 Overexpression of XB21 in XA21 plants (XB21ox/XA21) displays cell death lesions.
Kitaake wild type (Kit), transgenic rice carrying XA21 under the control of its native promoter (XA21, 23A-1-14), and XA21 plants overexpressing XB21 (XB21ox/Kit T1 lines, 2A-2, 3A-11, and 5A-2) were photographed at six-week stage.

## Slide 10
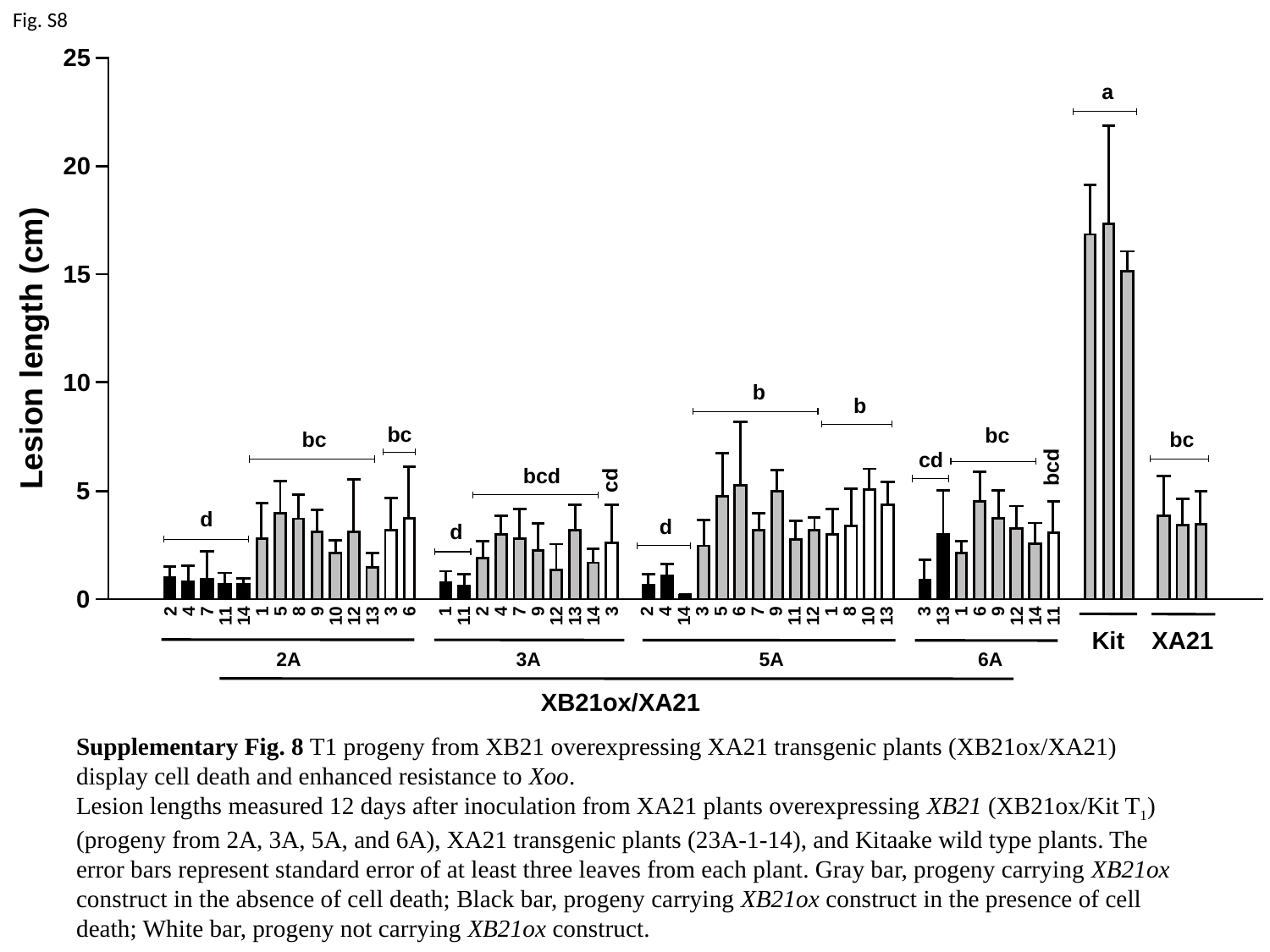

Fig. S8
25
a
20
15
Lesion length (cm)
10
b
b
bc
bc
bc
bc
cd
bcd
bcd
cd
5
d
d
d
0
2
4
3
5
6
7
9
1
8
2
4
7
1
5
8
9
3
6
1
2
4
7
9
3
3
1
6
9
13
14
11
12
10
11
14
10
12
13
11
12
13
14
13
12
14
11
Kit
XA21
2A
3A
5A
6A
XB21ox/XA21
Supplementary Fig. 8 T1 progeny from XB21 overexpressing XA21 transgenic plants (XB21ox/XA21) display cell death and enhanced resistance to Xoo.
Lesion lengths measured 12 days after inoculation from XA21 plants overexpressing XB21 (XB21ox/Kit T1) (progeny from 2A, 3A, 5A, and 6A), XA21 transgenic plants (23A-1-14), and Kitaake wild type plants. The error bars represent standard error of at least three leaves from each plant. Gray bar, progeny carrying XB21ox construct in the absence of cell death; Black bar, progeny carrying XB21ox construct in the presence of cell death; White bar, progeny not carrying XB21ox construct.

## Slide 11
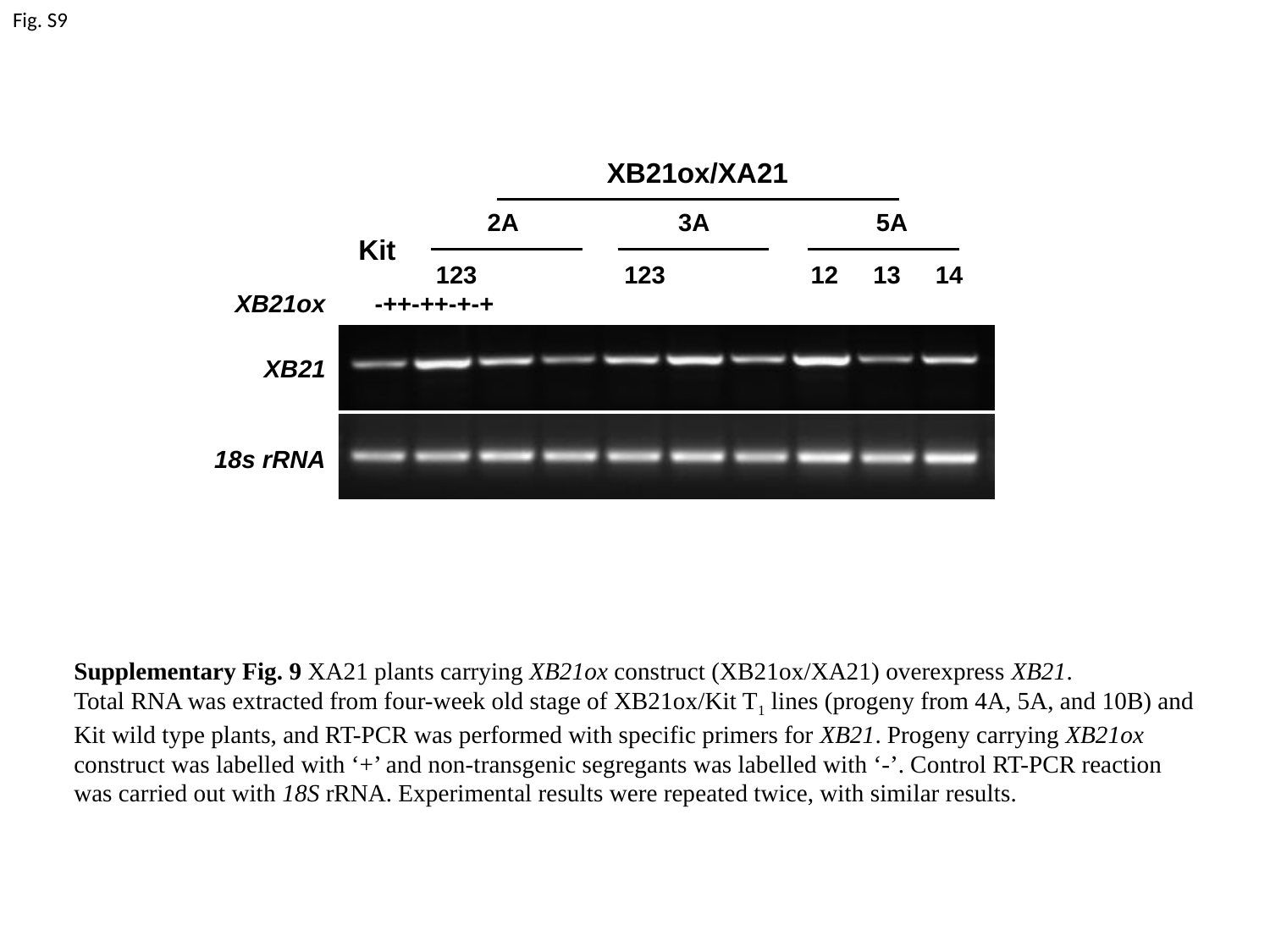

Fig. S9
XB21ox/XA21
2A 3A 5A
Kit
123
123
12 13 14
XB21ox
-++-++-+-+
XB21
18s rRNA
Supplementary Fig. 9 XA21 plants carrying XB21ox construct (XB21ox/XA21) overexpress XB21.
Total RNA was extracted from four-week old stage of XB21ox/Kit T1 lines (progeny from 4A, 5A, and 10B) and Kit wild type plants, and RT-PCR was performed with specific primers for XB21. Progeny carrying XB21ox construct was labelled with ‘+’ and non-transgenic segregants was labelled with ‘-’. Control RT-PCR reaction was carried out with 18S rRNA. Experimental results were repeated twice, with similar results.

## Slide 12
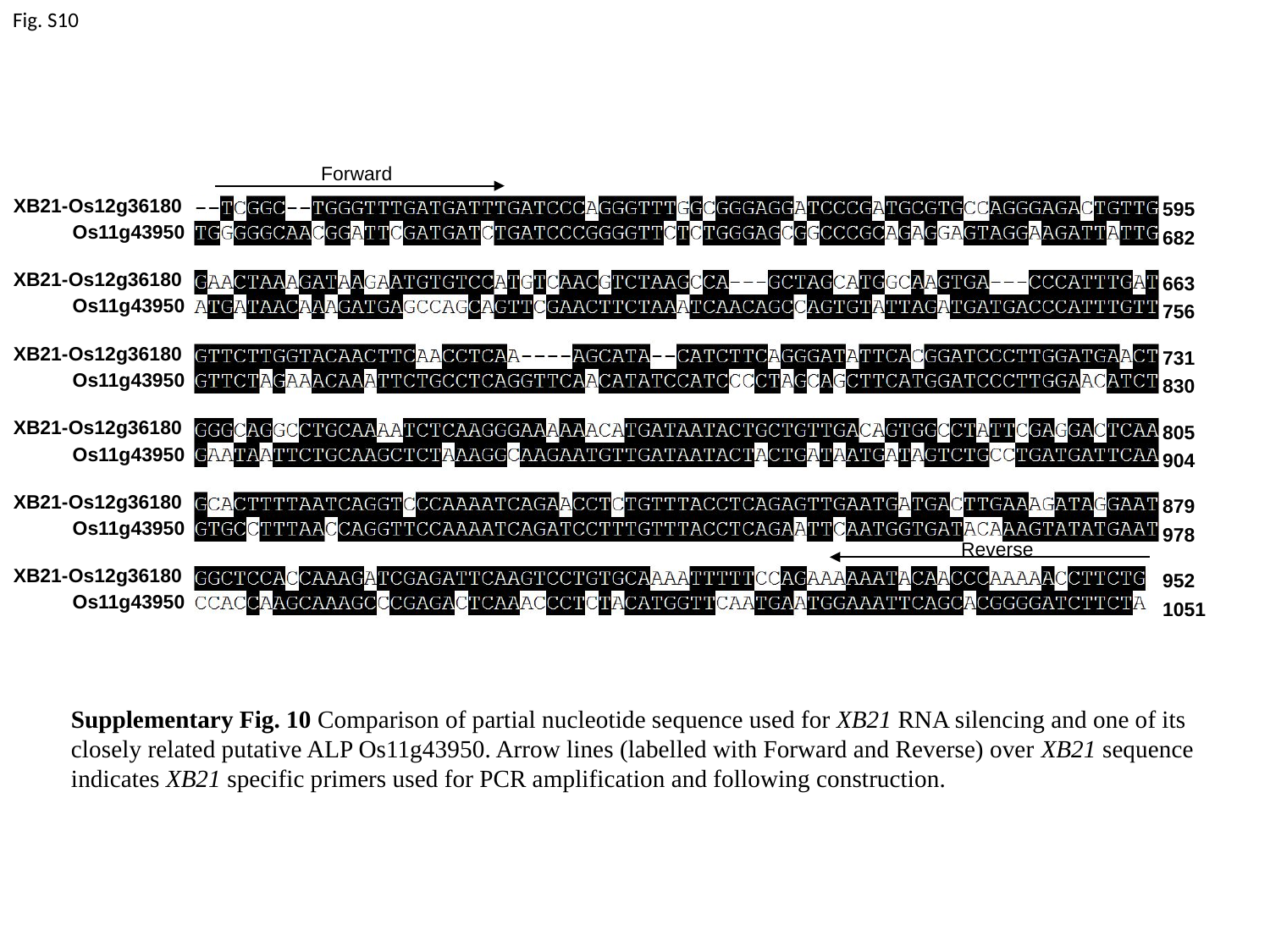

Fig. S10
Forward
595
682
XB21-Os12g36180
Os11g43950
663
756
XB21-Os12g36180
Os11g43950
731
830
XB21-Os12g36180
Os11g43950
805
904
XB21-Os12g36180
Os11g43950
XB21-Os12g36180
Os11g43950
879
978
Reverse
XB21-Os12g36180
Os11g43950
952
1051
Supplementary Fig. 10 Comparison of partial nucleotide sequence used for XB21 RNA silencing and one of its closely related putative ALP Os11g43950. Arrow lines (labelled with Forward and Reverse) over XB21 sequence indicates XB21 specific primers used for PCR amplification and following construction.

## Slide 13
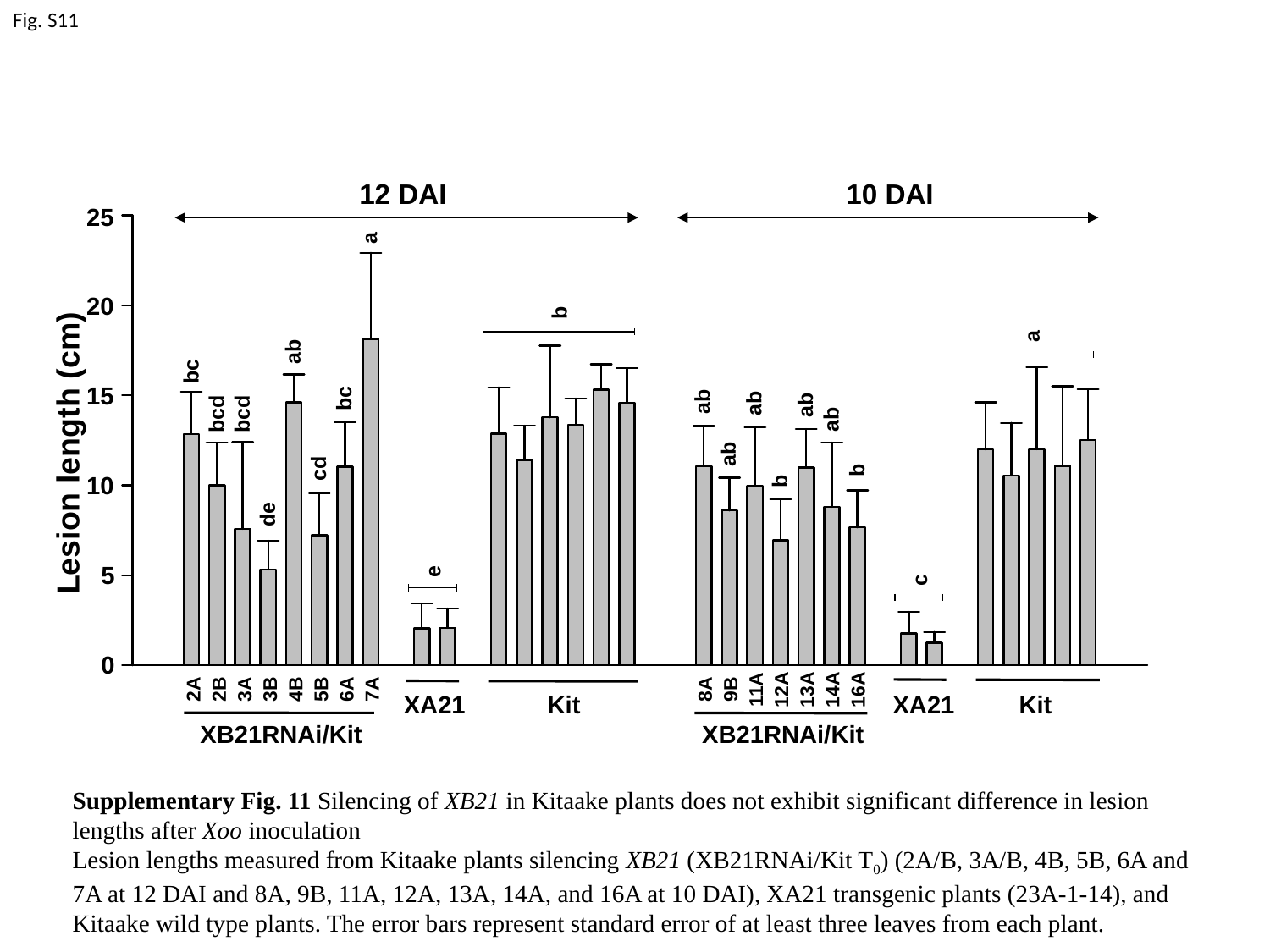

Fig. S11
12 DAI
10 DAI
25
a
20
b
a
ab
bc
15
bc
ab
ab
ab
bcd
bcd
ab
Lesion length (cm)
ab
cd
b
b
10
de
e
5
c
0
2A
2B
3A
3B
4B
5B
6A
7A
8A
9B
11A
12A
13A
14A
16A
XA21
Kit
XA21
Kit
XB21RNAi/Kit
XB21RNAi/Kit
Supplementary Fig. 11 Silencing of XB21 in Kitaake plants does not exhibit significant difference in lesion lengths after Xoo inoculation
Lesion lengths measured from Kitaake plants silencing XB21 (XB21RNAi/Kit T0) (2A/B, 3A/B, 4B, 5B, 6A and 7A at 12 DAI and 8A, 9B, 11A, 12A, 13A, 14A, and 16A at 10 DAI), XA21 transgenic plants (23A-1-14), and Kitaake wild type plants. The error bars represent standard error of at least three leaves from each plant.

## Slide 14
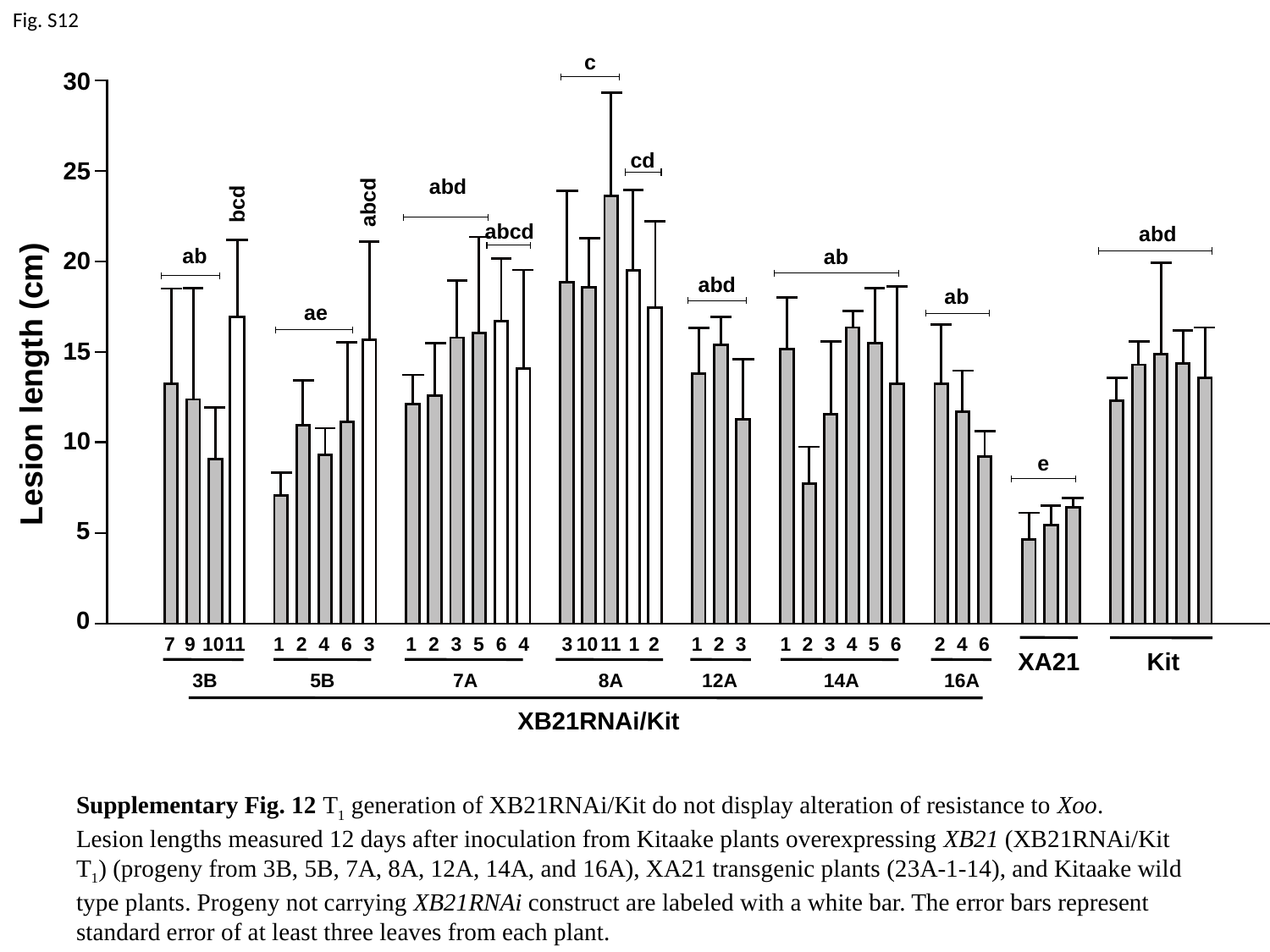

Fig. S12
c
30
cd
25
abd
abcd
bcd
abcd
abd
ab
ab
20
abd
ab
ae
15
Lesion length (cm)
10
e
5
0
7
9
10
11
1
2
4
6
3
1
2
3
5
6
4
3
10
11
1
2
1
2
3
1
2
3
4
5
6
2
4
6
XA21
Kit
3B
5B
7A
8A
12A
14A
16A
XB21RNAi/Kit
Supplementary Fig. 12 T1 generation of XB21RNAi/Kit do not display alteration of resistance to Xoo.
Lesion lengths measured 12 days after inoculation from Kitaake plants overexpressing XB21 (XB21RNAi/Kit T1) (progeny from 3B, 5B, 7A, 8A, 12A, 14A, and 16A), XA21 transgenic plants (23A-1-14), and Kitaake wild type plants. Progeny not carrying XB21RNAi construct are labeled with a white bar. The error bars represent standard error of at least three leaves from each plant.
